# Race and ethnicity in the COVID-19 Critical Care Consortium: demographics, treatments, and outcomes, an international observational registry study

**DOI:** 10.1186/s12939-023-02051-w

**Published:** 2023-12-12

**Authors:** Matthew J. Griffee, David A. Thomson, Jonathon Fanning, Dorothea Rosenberger, Adrian Barnett, Nicole M. White, Jacky Suen, John F. Fraser, Gianluigi Li Bassi, Sung-Min Cho, On behalf of the COVID-19 Critical Care Consortium, Heidi J. Dalton, John Laffey, Daniel Brodie, Eddy Fan, Antoni Torres, Davide Chiumello, Alyaa Elhazmi, Carol Hodgson, Shingo Ichiba, Carlos Luna, Srinivas Murthy, Alistair Nichol, Pauline Yeung Ng, Mark Ogino, Eva Marwali, Giacomo Grasselli, Robert Bartlett, Aidan Burrell, Muhammed Elhadi, Anna Motos, Ferran Barbé, Alberto Zanella

**Affiliations:** 1 Department of Anesthesiology, University of Utah School of Medicine, 30 N Mario Capecchi Drive, HELIX Tower 5N100, Salt Lake City, UT 84112 USA; 2 Department of Anaesthesia and Perioperative Medicine, Division of Critical Care, University of Cape Town, Cape Town, South Africa; 3 Critical Care Research Group, The Prince Charles Hospital, Chermside, Australia; 4 Faculty of Medicine, University of Queensland, Brisbane, QLD Australia; 5 Department of Anesthesiology, University Hospital Zurich, Zürich, Switzerland; 6 Australian Centre for Health Services Innovation and Centre for Healthcare Transformation, School of Public Health and Social Work, Queensland University of Technology, Brisbane, QLD Australia; 7 St Andrew’s War Memorial Hospital, UnitingCare, Spring Hill, QLD Australia; 8 Wesley Medical Research Foundation, Auchenflower, QLD Australia; 9 Wesley Hospital, Spring Hill, Auchenflower, QLD Australia; 10 Queensland University of Technology, Brisbane, Australia; 11 Departments of Neurology, Surgery, Anesthesia and Critical Care Medicine, Johns Hopkins Hospital, Baltimore, MD USA; 12 COVID-19 Critical Care Consortium, Principal Investigator, John Fraser, ECMOCARD@health.qld.gov.au, covid-critical.com, Critical Care Research Group, The Prince Charles Hospital, Metro North Hospital and Health Service, Clinical Sciences Building, Chermside, QLD 4032 Australia

**Keywords:** COVID-19, Respiratory distress syndrome, Healthcare disparities, American Indians or Alaska Natives, Race, Ethnicity, Structural racism

## Abstract

**Background:**

Improving access to healthcare for ethnic minorities is a public health priority in many countries, yet little is known about how to incorporate information on race, ethnicity, and related social determinants of health into large international studies. Most studies of differences in treatments and outcomes of COVID-19 associated with race and ethnicity are from single cities or countries.

**Methods:**

We present the breadth of race and ethnicity reported for patients in the COVID-19 Critical Care Consortium, an international observational cohort study from 380 sites across 32 countries. Patients from the United States, Australia, and South Africa were the focus of an analysis of treatments and in-hospital mortality stratified by race and ethnicity. Inclusion criteria were admission to intensive care for acute COVID-19 between January 14th, 2020, and February 15, 2022. Measurements included demographics, comorbidities, disease severity scores, treatments for organ failure, and in-hospital mortality.

**Results:**

Seven thousand three hundred ninety-four adults met the inclusion criteria. There was a wide variety of race and ethnicity designations. In the US, American Indian or Alaska Natives frequently received dialysis and mechanical ventilation and had the highest mortality. In Australia, organ failure scores were highest for Aboriginal/First Nations persons. The South Africa cohort ethnicities were predominantly Black African (50%) and Coloured* (28%). All patients in the South Africa cohort required mechanical ventilation. Mortality was highest for South Africa (68%), lowest for Australia (15%), and 30% in the US.

**Conclusions:**

Disease severity was higher for Indigenous ethnicity groups in the US and Australia than for other ethnicities. Race and ethnicity groups with longstanding healthcare disparities were found to have high acuity from COVID-19 and high mortality. Because there is no global system of race and ethnicity classification, researchers designing case report forms for international studies should consider including related information, such as socioeconomic status or migration background.

*Note: “Coloured” is an official, contemporary government census category of South Africa and is a term of self-identification of race and ethnicity of many citizens of South Africa.

**Supplementary Information:**

The online version contains supplementary material available at 10.1186/s12939-023-02051-w.

## Background

The COVID-19 pandemic overburdened healthcare systems with historic surges in hospital admissions, leading to resource limitations throughout the world. The strains on health systems magnified pre-existing social disparities. Consequently, vulnerable communities with longstanding health inequities faced additional challenges due to structural racism, poverty, lack of insurance, and other barriers to accessing high-quality healthcare [1, 2]. More information is needed about differences in critical care treatments and outcomes by race and ethnicity to highlight areas of healthcare disparities, and to guide quality improvement and outreach for vulnerable communities. The COVID-19 Critical Care Consortium (COVID Critical) is an international registry of patients with critical illness due to COVID-19. We examined demographics, treatments, and outcomes by race and ethnicity using COVID Critical data for three countries that had a relatively large mix of ethnicities.

## Methods

COVID Critical is a global, multicenter cohort study of 380 hospitals across 64 countries established to share data on patients with critical illness from COVID-19. The study design and protocol documents have been published previously [3]. Comorbidity definitions, as specified in the case report form (available at isaric.org), are provided in the Supplementary file. Participating sites obtained local ethics committee approval. Informed consent was waived based on the observational nature of the data and use of procedures to de-identify protected health information. Demographic and comorbidity data was collected at hospital admission and additional data fields were added throughout the hospital course, including treatments, complications, and outcomes.

### Study population

Patients admitted to an ICU between January 14th, 2020, and February 15, 2022 for treatment of COVID-19 were included. Only patients with a primary reason for ICU admission of COVID-19, per determination of site investigators, were eligible for enrollment. The majority of patients (> 90%) were confirmed to have COVID-19 by a PCR assay. (Patients without lab confirmation documentation were included in the analysis because sites with limited resources were included.) Exclusion criteria were missing admission date; patients from countries (Spain and Italy) where ethnicity was not collected; and patient request to not disclose ethnicity. Patients for whom the case report form included the ethnicity field, but it was unanswered, were included; patients without any ethnicity question field in the demographics form were excluded. Inclusion requirements were diagnosis of acute COVID-19 and admission within the query dates.

### Description of race and ethnicity

The framework of the American Medical Association Manual of Style Committee “Updated Guidance on the Reporting of Race and Ethnicity in Medical and Science Journals” was used for this study [4]. For brevity, we predominantly use “ethnicity” to stand for “race and ethnicity.” The first objective was to describe ethnicity across the entire international cohort using the categories provided in the case report form (Appendix 1). Site investigators at each hospital determined the ethnicity of patients. The methods of determining ethnicity endorsed most frequently by site investigators were “review of available data,” “based on demographics in the medical record,” and “based on a patient’s self-identification or family/surrogate interview”.

### Treatments and outcomes

The second objective was to describe the frequency of selected ICU treatments, including mechanical ventilation and dialysis, by ethnicity. The final objective was to compare in-hospital mortality and length of stay by ethnicity. For evaluating treatment and outcomes by ethnicity, we focused on three countries: the United States (US), Australia, and South Africa. These countries were selected based on adequate patient numbers and a diversity of ethnicity groups. In the analysis of treatments and outcomes, the race and ethnicity categories of the country’s government census were used (rather than the original ethnicity categories of the case report forms).

### Statistical analysis

We used descriptive statistics to examine differences in patients’ characteristics by ethnicity, using tables of percentages for categorical variables, and graphical summaries using boxplots for continuous variables. We used time-to-event models to examine differences in the patients’ journeys after admission to hospital. We examined the time to discharge or death, and censored patients who were still in hospital at the end of data collection (Supplementary Fig. 1, Model 1). Examining time to discharge is akin to examining length of stay. We examined the time to invasive mechanical ventilation with death and discharged alive as competing risks (Supplementary Fig. 1, Model 2) [5]. We examined the time to the first event, so we did not examine the time from mechanical ventilation to death and discharge. We used cumulative probability curves to graphically compare the differences between ethnicity groups. The competing risks of ventilation, survival to discharge, and death meant we could not use Kaplan–Meier curves [6]. We used Weibull survival models to examine differences by ethnicity in the hazards of death, discharge, and mechanical ventilation. The Weibull survival model was selected because it has a simple parametric function for the hazard, compared with the Cox model. We visualized the estimated parametric hazard, and checked for large residuals. We used a Bayesian approach and fitted ethnicity as a random effect with a reference group of the overall average. We plotted the hazard ratios by ethnicity group and the 95% credible intervals.

We compared average Acute Physiology and Chronic Health Evaluation (APACHE) II scores between ethnicity groups using a Bayesian model. We used a regression model with ethnicity as the independent variable and calculated the posterior probability that the mean for each ethnicity was greater than the overall average. We prefer these Bayesian posterior probabilities as they are easier to interpret than *p*-values [7]. Our main results examined the association between ethnicity and the outcomes of in-hospital mortality and length of stay, without adjusting for other patient characteristics. In a second model, we adjusted for age and site.

In a sensitivity analysis, we allowed the effect of ethnicity to vary by site using a site-specific effect for ethnicity in a random effects model. The aim was to determine whether variability from site to site accounted for any differences between ethnicity groups in the risks of mortality or time to discharge. We used R (version 4.2.0) for database management and plots [8], and INLA (version 22.03.16) to fit the Bayesian models [9].

## Results

### Ethnicity of patients in the COVID critical international registry

**Fig. 1:**
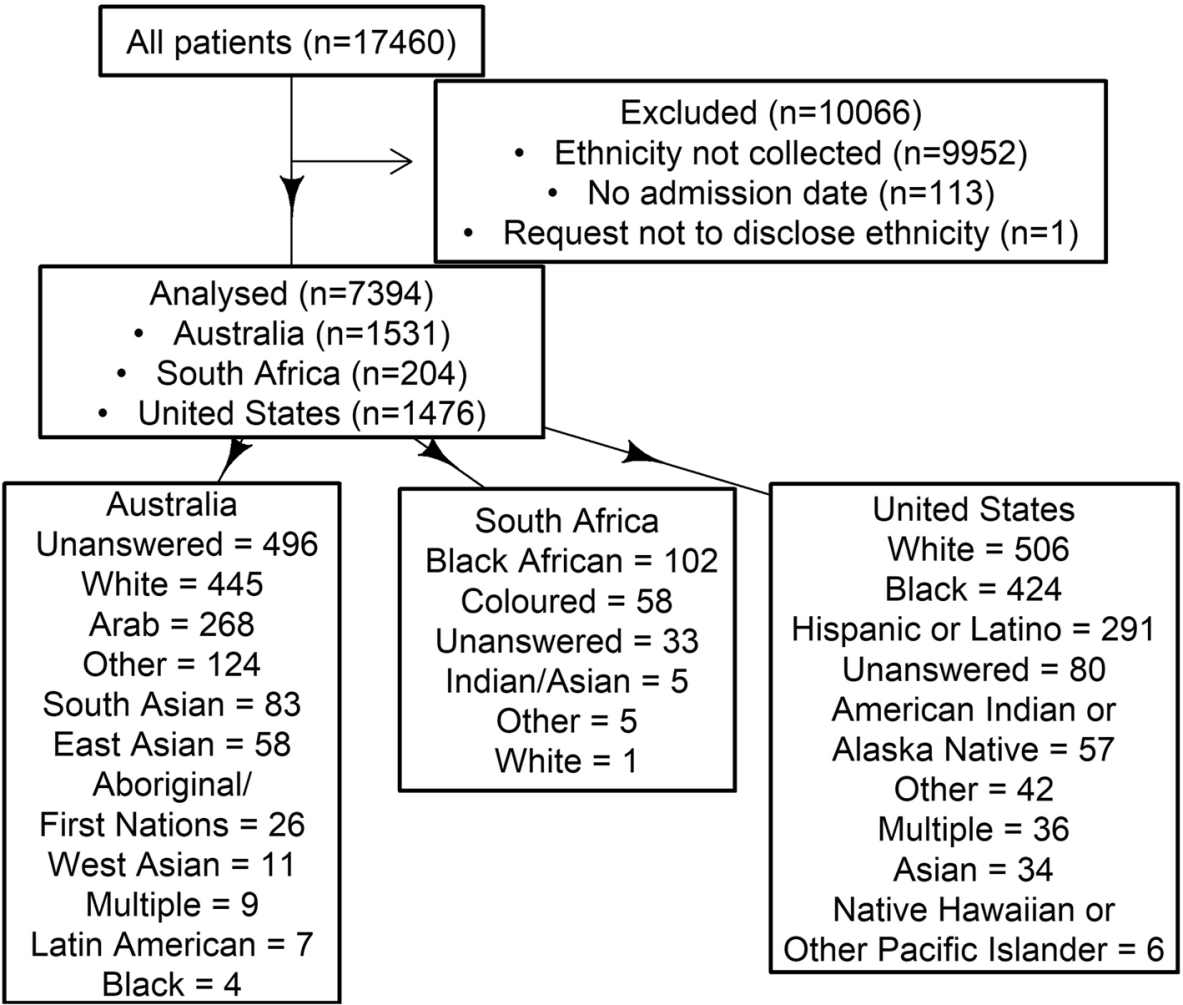
Patient flow diagram, with cohort counts and outcome statistics for Australia, South Africa, and the United States

**Fig. 2:**
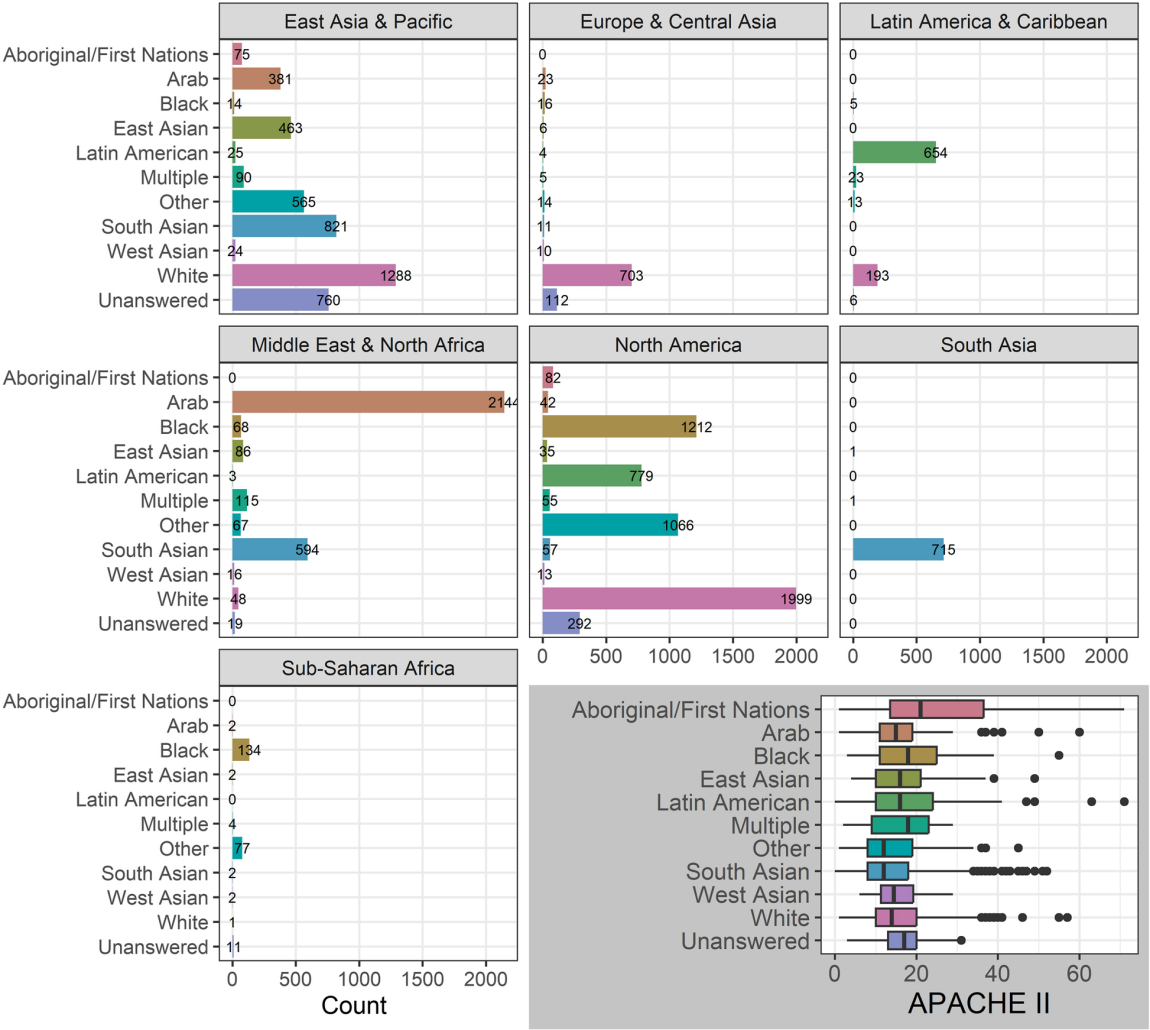
Patient counts by ethnicity across World Bank Regions. Histograms of patient counts by ethnicity for the seven World Bank Regions. INSET: APACHE II scores by ethnicity box plots. The rectangular box extends from the first to the third quartile. The line in the box is the median score. The upper and lower whiskers extend from the box to the largest and smallest scores, no further than 1.5 inter-quartile ranges from the box. Scores beyond the end of the whiskers are outlying points and are plotted individually

Overall, 7,394 patients from 32 countries met the inclusion criteria (Fig. 1, Tables 1 and 2). 10,066 patients had case report forms which did not include ethnicity in the demographics. 114 patients were excluded as they had no admission date. Demographics, treatments, and outcomes of the international cohort are presented in Table 1; the countries and patient counts per country are presented in Table 2. 92% of patients had documentation of lab confirmation of COVID-19, the vast majority with a PCR assay. Reasons for missing laboratory confirmation include limited resources for patients in low- and middle-income countries and incomplete records of patients initially diagnosed at a hospital which transferred the patient to a center participating in the registry. As specified in the methods, only patients with a primary reason for ICU admission of COVID-19 were eligible for enrollment.The diversity of ethnicity categories varied substantially by World Bank Region (Fig. 2). No response was entered into the ethnicity field in 13% (coded as “Unanswered”), a single ethnicity was selected for 84%, and two or more ethnicities were selected for 2.9% of patients (coded as Multiple). White was the most frequent ethnicity among high-income countries; South Asian was the most frequent ethnicity among low- and middle-income countries. The “Other” category was selected for the ethnicity of 540 patients (7.3% of the total). The percentage of patients for whom “Other” was selected varied from 0% (for 21 countries) to > 30% (for Indonesia and South Africa). When “Other ethnicity” was selected, investigators were asked to complete a free text response. There were 68 unique identity terms defined for this response (Appendix 2). Some of the “Other ethnicity” patients were mapped into the standard categories: for example, the patient with free text “Native American Indian” was moved into the category American Indian or Alaska Native.

**Table 1:** Demographic table for whole cohort, excluding countries that did not collect ethnicity. The Missing column shows the percentage missing. Q1 and Q3 are the first and third quartiles for continuous variables

	Overall	Missing
n	7,394	
Age, Median [Q1, Q3]	57 [45, 67]	1.9
Male, n (%)	4686 (65)	1.9
APACHE_II, Median [Q1, Q3]	14 [10, 20]	75
SOFA, Median [Q1, Q3]	5 [3, 8]	78
BMI, Median [Q1, Q3]	27.7 [24.0, 33.2]	40
Lab confirmed COVID19, n (%)	5791 (92)	15
Outcome, n (%)		0
Unknown	1074 (15)	
Death	2230 (30)	
Discharged alive	2350 (32)	
Discharged home	842 (11)	
Hospitalization	250 (3.4)	
Palliative discharge	27 (0.4)	
Transfer to another facility (rehab)	88 (1.2)	
Transfer to other facility	440 (6.0)	
Transfer to other facility (acute hospital)	93 (1.3)	
Comorbidities		
Chronic kidney disease, n (%)	631 (9.4)	9.2
Diabetes, n (%)	2232 (34)	11
Hypertension, n (%)	2672 (47)	23
Liver disease, n (%)	169 (2.5)	9.1
Malnutrition, n (%)	112 (1.7)	9.5
Obesity, n (%)	1980 (30)	9.8
Smoking, n (%)	1278 (19)	9.1
Treatments		
High flow nasal oxygen, n (%)	972 (41)	68
Noninvasive ventilation, n (%)	1279 (28)	37
Invasive ventilation, n (%)	3051 (65)	37
Extracorporeal support, n (%)	690 (15)	37

**Table 2:** Patient counts by country

**Income**	**Country**	**N**	**Percent**
HIC	Australia	1,531	29.0
	Austria	52	1.0
	Belgium	93	1.8
	Canada	242	4.6
	Chile	138	2.6
	Estonia	146	2.8
	Germany	121	2.3
	Hong Kong	20	0.4
	Ireland	206	3.9
	Japan	247	4.7
	Kuwait	525	9.9
	Netherlands	11	0.2
	Poland	15	0.3
	Portugal	16	0.3
	Qatar	154	2.9
	Saudi Arabia	21	0.4
	Singapore	1	0.0
	South Africa	204	3.8
	South Korea	44	0.8
	Taiwan	1	0.0
	United Arab Emirates	41	0.8
	United States	1,477	28.0
LMIC	Argentina	131	6.3
	Brazil	89	4.3
	China	12	0.6
	Colombia	356	17.0
	India	594	28.0
	Indonesia	843	40.0
	Mexico	4	0.2
	Peru	50	2.4
	Thailand	9	0.4
	Vietnam	1	0.0

Countries included

The table shows the countries included in the analysis. The first column shows whether the country is high or low/middle income

### Disease severity by ethnicity (international cohort)

APACHE II scores had a wide overlap across ethnicity groups, except for Aboriginal/First Nations, which had a higher mean than other groups (posterior probability of higher than the average mean of 1.0, Fig. 2, inset).

### Treatments and outcomes- COVID critical United States (US) cohort

#### Demographics and comorbidities

The ethnicity categories for the US cohort were adapted from the US Census (https://www.census.gov/topics/health/data.html). White, Black, and Hispanic or Latino accounted for the majority of the 1,476 patients, with the other ethnic groups composing under 5% each (Supplementary Fig. 2A). The prevalence of diabetes was higher for Black and American Indian or Alaska Native persons than for other groups (Supplementary Fig. 3).

#### Disease severity

The APACHE II scores were similar for all ethnicities except for American Indian or Alaska Natives, who had a score higher than the overall average (posterior probability of higher mean 1.0, Supplementary Fig. 4).

#### Treatment frequency by ethnicity

Treatments for respiratory failure, provision of dialysis, and use of ECMO during the ICU course varied substantially by ethnicity in the US cohort (Table 3). The groups with the highest frequency of dialysis were American Indian or Alaska Native and Native Hawaiian or Other Pacific Islander (42% and 40%, respectively), and the groups with the highest frequency of invasive mechanical ventilation were other ethnicity and American Indian or Alaska Native (90% and 89%, respectively).

**Table 3:** Treatment during hospitalization, US cohort. Cells show the percentage and numerator / denominator in round brackets

	**Dialysis**	**ECMO**	**HF Nasal Oxygen**	**Invasive Mechanical Ventilation**	**Non-invasive Mechanical Ventilation**
American Indian or Alaska Native	42% (24/57)	23% (13/57)	91% (52/57)	89% (51/57)	49% (28/57)
Asian	20% (5/25)	34% (10/29)	64% (16/25)	84% (21/25)	60% (15/25)
Black	28% (107/381)	22% (83/381)	61% (228/371)	63% (241/382)	41% (156/379)
Hispanic or Latino	23% (51/220)	43% (100/231)	71% (156/220)	79% (175/221)	40% (87/220)
Native Hawaiian or Other Pacific Islander	40% (2/5)	50% (3/6)	60% (3/5)	80% (4/5)	80% (4/5)
Unanswered	22% (15/69)	32% (22/69)	51% (34/67)	77% (53/69)	29% (20/69)
Other	34% (10/29)	52% (16/31)	41% (12/29)	90% (26/29)	43% (12/28)
White	26% (118/457)	23% (105/462)	64% (287/449)	72% (332/459)	44% (203/457)

Treatments during hospitalization, US Cohort, by ethnicity. The cells show the percentage of patients who had the specified treatment at any point in their hospital stay. The numerator and denominator under each percentage are the number of patients who had the treatment and the number of patients who had a yes or no response in the case report form for the designated treatment, respectively

#### In-hospital mortality by ethnicity, US cohort

**Fig. 3:**
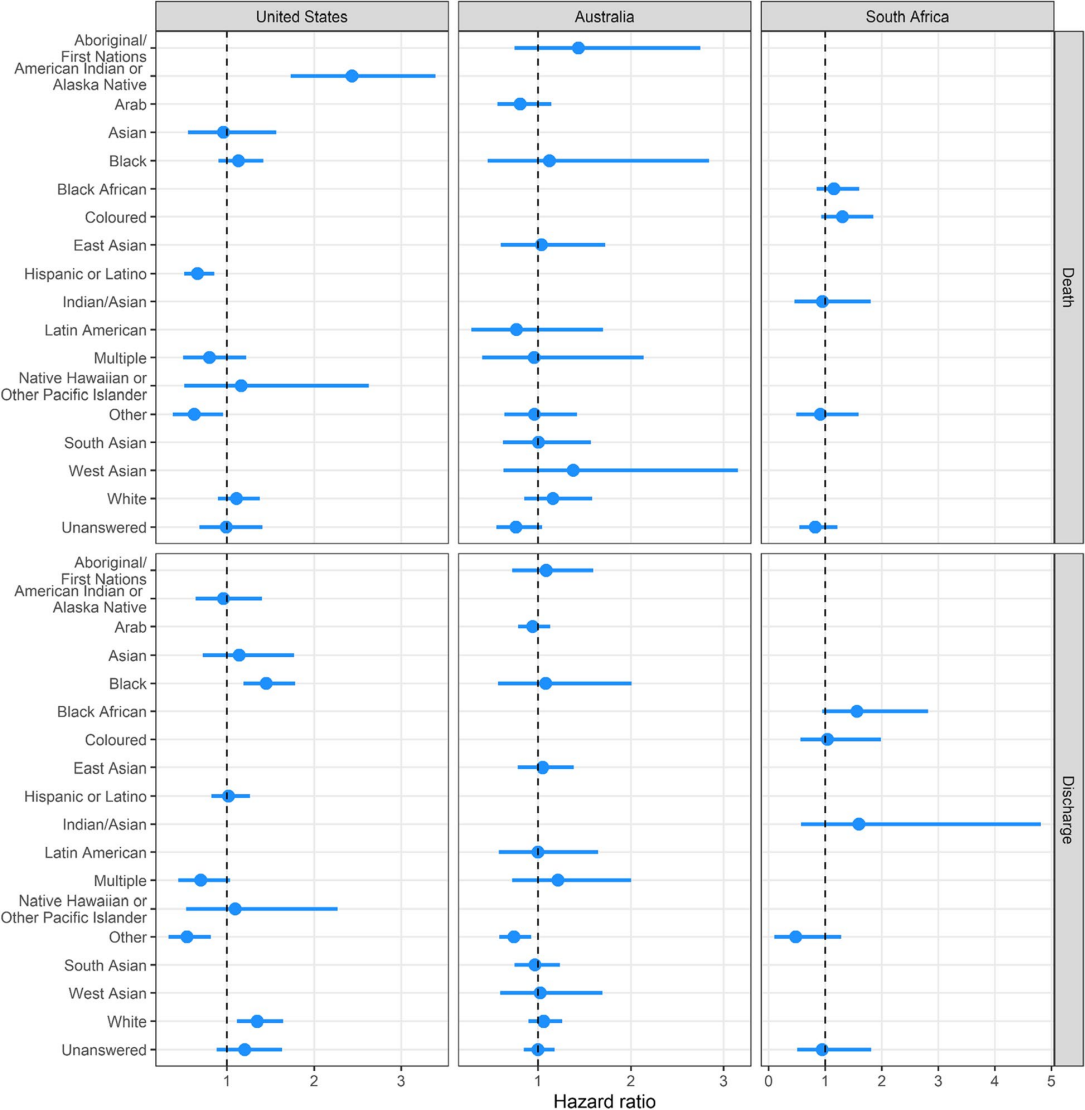
Hazard ratio for in-hospital mortality and discharged alive by ethnicity, US, Australian, and South African cohorts. The blue dot is the hazard ratio for death (upper three panels) and discharged alive (lower three panels) and the blue whiskers are the 95% credible intervals. Higher hazard of discharged alive indicates shorter length of stay. The reference line of 1 in the hazard plots is for the overall average for all patients

**Fig. 4:**
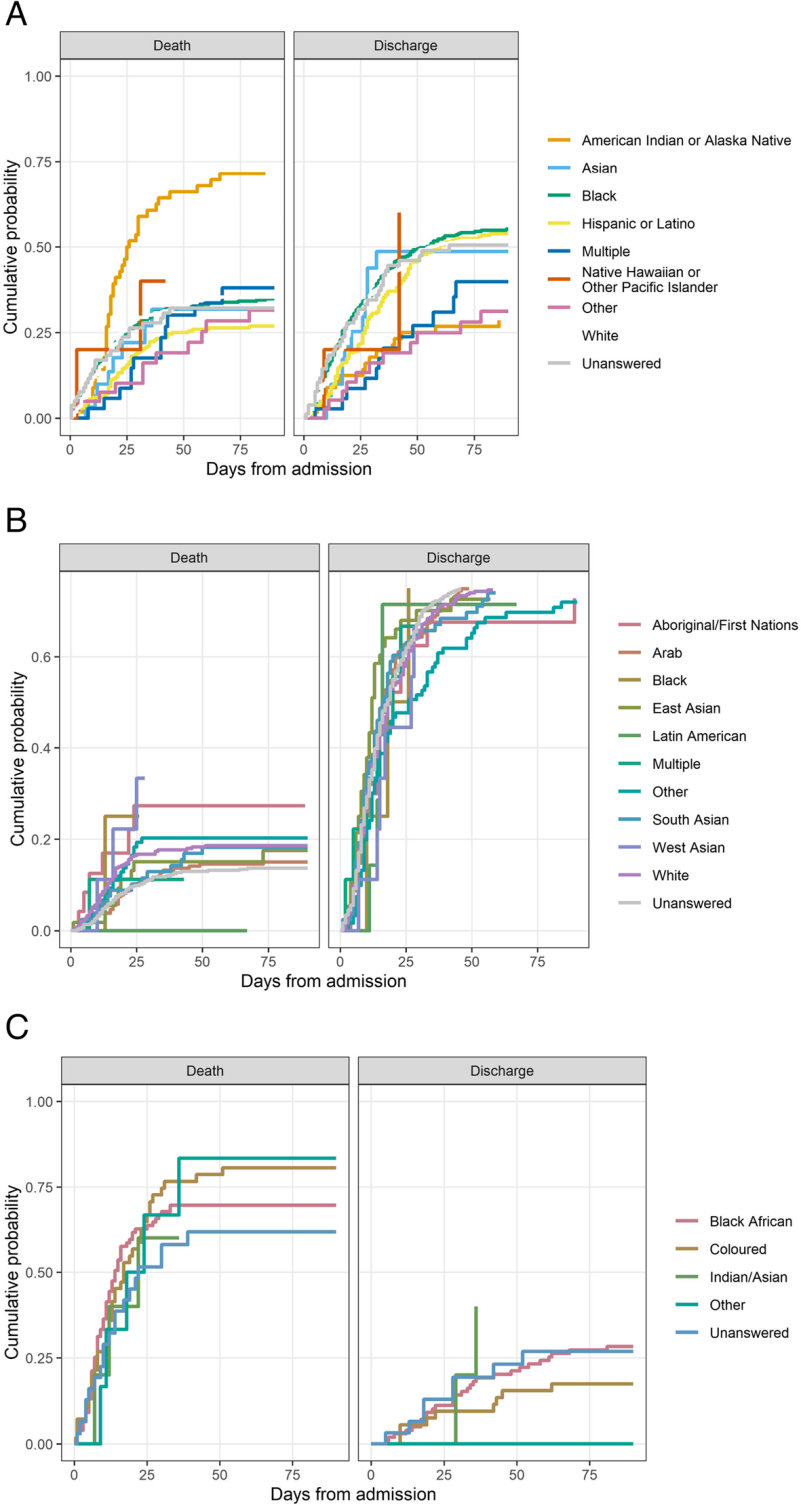
Cumulative probability of mortality (left panels) and discharged alive (right panels) for US (**A**), Australian (**B**), and South African (**C**) cohorts. The x-axis represents days from admission, the y-axis the cumulative probability of the outcome. Ethnicity categories are color-coded according to legends at the right side

In the primary survival analysis, the hazard of death was increased for American Indian or Alaska Native (HR 2.43; 95% CI, 1.73 to 3.39) and decreased for Hispanic or Latino (HR 0.66; 95% CI, 0.51 to 0.86), (Fig. 3). The cumulative probability of death was higher for American Indian or Alaska Natives than for other ethnicities (Fig. 4A and Supplementary Fig. 5A). After adjusting for age and site, the risk of death for American Indian or Alaska Native persons was still elevated (HR 1.29; 95% CI, 0.91–1.9), but the confidence interval included 1.0 (Supplementary Fig. 6A). There was a persistent increased risk of death for American Indian or Alaska Native persons when there was adjustment for age alone, but not when there was adjustment for site alone (Supplementary Fig. 6B, C).

A sensitivity analysis evaluated the uniformity of outcomes across sites, comparing a model treating the effect of ethnicity across sites as a fixed effect to a model treating the effect of ethnicity across sites as a random effect. The fit for both the survival model and the discharged alive model by ethnicity was better when the effect of ethnicity varied by site. There was significant variation by site in the risk of death for seven out of nine ethnicity groups (Supplementary Fig. 7).

### Treatments and outcomes- COVID critical Australian cohort

#### Demographics and comorbidities

The cohort size was 1531 patients, from 13 sites, with the median number per site of 88. The original ethnicity categories of the case report form were retained, and three additional categories were added into the options: Unanswered, Other, and Multiple ethnicities (Supplementary Fig. 2B). The largest ethnicity groups were Unanswered (31%), White (30%), and Arab (18%), with all other groups composing under 10%. Only 1% of patients had two categories selected. Diabetes, Obesity, and Smoking were more frequent for Aboriginal/First Nations and West Asians than for other groups (Supplementary Fig. 8).

#### Disease severity

SOFA scores were lower for the East Asian and Latin American groups than average (posterior probability of score being lower than the overall average 0.96 and 0.86, respectively), and SOFA scores were higher for Aboriginal/First Nations persons than average (posterior probability of score being higher than overall average, 0.79, Supplementary Fig. 9). APACHE II scores were not reported for the Australian cohort.

#### Treatment frequency by ethnicity

Mechanical ventilation was used frequently, with variation across ethnicities (Table 4). Renal replacement therapy, ECMO, and non-invasive mechanical ventilation were used infrequently (Table 4).

**Table 4:** Treatment during hospitalization, Australian cohort

	Dialysis	ECMO	High-flow nasal Oxygen	Invasive Mechanical Ventilation	Non-invasive Mechanical Ventilation
Aboriginal/First Nations	29% (2/7)	0% (0/1)	57% (4/7)	71% (5/7)	14% (1/7)
Arab	6% (4/64)	6% (1/17)	68% (34/50)	74% (37/50)	18% (9/50)
Black	100% (1/1)	100% (1/1)	0% (0/1)	100% (1/1)	0% (0/1)
East Asian	5% (1/21)	11% (2/19)	43% (9/21)	38% (8/21)	14% (3/21)
Latin American	0% (0/2)	0% (0/2)	0% (0/2)	0% (0/2)	0% (0/2)
Unanswered	19% (9/47)	12% (3/24)	58% (25/43)	63% (27/43)	16% (7/43)
Other	9% (2/22)	8% (1/12)	68% (13/19)	71% (15/21)	21% (4/19)
South Asian	10% (2/21)	9% (1/11)	68% (13/19)	95% (18/19)	5% (1/19)
West Asian	0% (0/2)	0% (0/0)	100% (2/2)	100% (2/2)	0% (0/2)
White	10% (6/61)	5% (2/40)	54% (30/56)	68% (39/57)	11% (6/56)

#### Risk of in-hospital mortality by ethnicity

In the primary mortality analysis, with ethnicity as an independent variable, the hazard ratio varied from 0.76 to 1.44, with all confidence intervals overlapping the identity line (Fig. 3). Unanswered ethnicity and Latin Americans had the lowest risk of death (HR, 0.76, 95% CI 0.55 to 1.05, and HR, 0.77, 95% CI 0.28 to 1.70, respectively). Aboriginal/First Nations persons had the highest risk of death (hazard ratio, 1.44, 95% CI 0.75 to 2.75) followed by West Asian persons (hazard ratio, 1.38, 95% CI, 0.63 to 3.15). The credible interval widths varied widely due to the differences in patient numbers. Cumulative mortality was highest for the ethnicities West Asian and Aboriginal/First Nations (Fig. 4B).

#### Risk of discharge by ethnicity

In the unadjusted analysis, the other ethnicity cohort had a lower hazard of discharged alive (HR, 0.74; 95% CI, 0.58 to 0.93), corresponding to a longer length-of-stay (Fig. 3 middle panel, Supplementary Fig. 5B). (Note that a favorable course, namely, a shorter length-of-stay, would correspond to a *higher* hazard of reaching the end-point alive at discharge in the model.) The decreased hazard of discharged alive for other ethnicity persisted when outcomes were adjusted for age and site (HR 0.76; 95% CI, 0.60 to 0.95).

The fit for both the survival model and the discharged alive model by ethnicity was better with a model including a site-specific effect for ethnicity. In the sensitivity analysis, there were a few sites with statistically significant variation in length of stay by ethnicity (Supplementary Fig. 10A). In the evaluation of variation in the hazard of death by site and ethnicity, four ethnicity-site pairings had significantly increased risk (Supplementary Fig. 10B).

### Treatments and outcomes- COVID critical South African cohort

South Africa had a single contributing site. Of 204 patients meeting inclusion criteria, 50% were Black African, 28% were Coloured, and 16% were in the Unanswered ethnicity groups (Supplementary Fig. 2C).

Disease severity by SOFA score was higher for Indian/Asian ethnicity patients (posterior probability of higher than the average mean, 0.83, Supplementary Fig. 11). AIDS/HIV was present in 20% of the Black African ethnicity patients and 6% of the Unanswered ethnicity patients.

Although uncommon overall, dialysis use by ethnicity varied substantially (Table 5). 100% of patients (of all ethnicities) had invasive mechanical ventilation (Table 5). In the survival analysis, the risk of death was similar across ethnicity groups (Figs. 3 and 4C). Cumulative mortality was greater than 50% for all ethnicities and greater than 75% for the Coloured and Other ethnicity groups (Fig. 4C and Supplementary Fig. 5C). 2

**Table 5:** Treatment during hospitalization, South African cohort

	Dialysis	ECMO	High Flow Nasal Oxygen	Invasive Mechanical Ventilation	Non-invasive Mechanical Ventilation
Black African	13% (13/102)	1% (1/102)	73% (74/102)	100% (102/102)	21% (21/102)
Coloured	16% (9/58)	3% (2/58)	83% (48/58)	100% (58/58)	22% (13/58)
Indian/Asian	60% (3/5)	0% (0/5)	60% (3/5)	100% (5/5)	20% (1/5)
Unanswered	3% (1/32)	0% (0/30)	80% (24/30)	100% (32/32)	7% (2/30)
Other	20% (1/5)	0% (0/5)	40% (2/5)	100% (5/5)	40% (2/5)
White	100% (1/1)	0% (0/1)	100% (1/1)	100% (1/1)	0% (0/1)

## Discussion

We investigated the ethnicity designation of 7,394 critically ill patients with COVID-19 from 32 countries and observed wide variations in the frequency with which the ethnicity question was answered and in the range of ethnicity categories selected. Because the definition of ethnicity depends on specific cultural contexts, there is no coherent international classification system. This is a potential explanation for the number of patients with ethnicity unanswered or designated “Other”. Adding additional options pertaining to social determinants of health to case report forms, such as socioeconomic status, education level, insurance coverage, and refugee status may improve response rates and clarify potential reasons for healthcare disparities by ethnicity [10]. Joint analysis of socioeconomic status and ethnicity may provide insight to clinicians working to improve equity in healthcare [11].

Evaluation of differences in treatments and outcomes of severe COVID-19 by ethnicity is clinically relevant: we found that ethnicity was associated with significant differences in disease severity, critical care treatments, length of stay, and in-hospital mortality. Practical steps for clinicians to consider to improve equity across ethnicity groups include tracking local hospital performance (treatments and outcomes) by ethnicity; prioritizing vaccination programs for vulnerable communities; and inclusion of tribal and minority leaders in customizing public health recommendations to decrease the spread of COVID-19 in culturally sensitive ways- particularly for communities with limited information technology [12, 13]. Critical care providers who identify ethnicity groups with high rates of mechanical ventilation may consider optimizing strategies to avoid the need for intubation, e.g., proning and early use of non-invasive ventilation.

The clinical importance of benchmarking site-specific metrics of outcomes by ethnicity emerges from the sensitivity analyses, showing that variability in outcomes (both for mortality and length-of-stay) are influenced by sites with significant differences in performance (Supplementary Figs. 7 and 10A and B). Moreover, the increased mortality of American Indian or Alaska Native persons was not statistically significant when adjusted for site (Supplementary Fig. 6C). Sites with worse outcomes for particular ethnicity groups may benefit from dialogue with sites with more equitable outcomes to identify strategies for overcoming barriers to accessing advanced care, to improve the quality of care for vulnerable groups.

Inequalities which are delineated according to ethnicity categories can be identified only if the research design incorporates the relevant demographic information. Guidance for including race and ethnicity (and related terms) in case report forms for international registry research is not readily available in the medical literature. Adaptation of race and ethnicity categories to be relevant for country-specific concepts of race and ethnicity may be useful. We propose a framework that specifies use of local ethnicity designations, emphasizes self-definition, and includes relevant, related socioeconomic designations (Appendix 4).

### Ethnicity differences in risk of death

In this global registry, Aboriginal/First Nations patients (comprised of persons from the US, Canada, and Australia) had higher average disease severity scores compared to the scores of other ethnicity groups, and in the US cohort, the American Indian or Alaska Native APACHE II score was also significantly higher than the overall average. 89% of patients in the American Indian or Alaska Native group required invasive mechanical ventilation. In addition to a high rate of mechanical ventilation, American Indian or Alaska Native persons had the highest frequency of dialysis in the US cohort, suggesting presentation at a phase of illness with multisystem organ failure (Table 3). In the primary mortality analysis, the American Indian or Alaska Native cohort had a significantly higher risk of death than average. These findings reinforce studies that have demonstrated stark healthcare disparities in outcomes of COVID-19 for persons of American Indian or Alaska Native heritage [14–16]. For some patients who rely on the Indian Health Service, geographical challenges of transportation from remote rural areas to urban hospitals may account for presentation at an advanced stage of illness [17]. Although the registry did not include information on healthcare insurance, it is noteworthy that there are many challenges for some American Indian or Alaska Native persons who rely on the Indian Health Service to access advanced resources (such as ICU) [18]. Likewise, it should be noted that private insurance coverage is more common among White than among non-White South Africans, and most of the South African cohort was non-White (Appendix 3). These examples illustrate the potential importance of insurance coverage as a factor reflecting socioeconomic status which may correlate with presentation of COVID-19 at advanced stages and with poor outcomes- and call for insurance coverage to be included in demographics in future registries..

Our finding of a decreased risk of death for Hispanic or Latino persons admitted to ICU in the US contradicts other studies [19, 20]. The cohort of Hispanic or Latino patients in our study had a lower disease severity score than the average for the US cohort, and the decreased risk of in-hospital mortality was not present after adjustment for site. One site had a particularly low hazard of death for Hispanic or Latino persons (Supplementary Fig. 7). The study of Acosta et al. used a population-based cross-sectional study design, and showed a higher risk of hospitalization, ICU admission, and death for Latino persons with COVID-19 compared to White persons [20]. The finding that disease severity was lower than average for the Hispanic or Latino cohort in this study suggests that a substantial number of these patients may have received care in hospitals where patients with relatively low acuity are admitted to the ICU.

### South African COVID critical outcomes

The South African cohort reflects the experience of patients from a single public center. 100% of patients required mechanical ventilation and all ethnic groups in the South African cohort had mortality well over 50%, exceeding the mortality of all ethnicities in the Australian cohort and of all ethnicities in the U.S. cohort, with the exception of American Indian or Alaska Native persons (Fig. 4 and Supplementary Fig. 5A and C). A high frequency of mechanical ventilation early in the course of critical care was similar for the South African cohort and for the American Indian or Alaska Native cohort (Supplementary Fig. 12A and C). The high mortality for these cohorts was potentially a reflection of delayed presentation, which may derive from disparities in access to ICU resources.

The ethnic composition of the population, economic resources, and access to private healthcare insurance vary substantially across South Africa, resulting in dramatic differences in access to hospitals and to ICU care from one area to another [21]. Health and socioeconomic disparities which have roots in a legacy of apartheid correlate with shorter life expectancy in areas with larger non-White demographics in South Africa ([21–23], Appendix 3). The South African COVID-19 surveillance program demonstrated that in-hospital mortality from severe COVID-19 was higher for Black, Coloured, and Indian descent patients than for White patients [23].

Some terminology of ethnicity categories from the South African cohort highlights that the nomenclature of ethnicity cannot be assumed to have the same meaning from one country to another. For example, the “Coloured” ethnicity description of South Africa identifies persons with descent from both Indigenous South African Khoisan ancestors and European and African migrants to the area over the past 350 years [24].

### Ethnicity in the Australian COVID critical cohort analysis

Although not reaching statistical significance, the hazard of death was highest for Aboriginal/First Nations and West Asian ethnicity groups, which had higher SOFA scores and an elevated prevalence of some comorbidities, compared to the other ethnicity groups. Cumulative mortality was higher for these ethnicity groups than for other ethnicity groups. A higher prevalence of metabolic syndrome comorbid conditions (namely, obesity and diabetes) noted for the ethnicity groups with the highest cumulative mortality may indicate disparities in preventive health and healthcare maintenance... In particular, comorbidities related to malnutrition may be a legacy of effects of a colonial food system for Aboriginal persons in Australia [25, 26].

### Strengths and limitations of the study

Studies on ethnicity, COVID-19, and healthcare disparities have derived predominantly from single cities, individual countries, and a limited number of ethnicity groups [1, 2, 10, 12, 16, 19, 20, 23, 27]. The COVID Critical registry provided international data. A potential explanation for the lower mortality for the Australian patients than for the US patients and South African patients is that most enrollment in Australia was later in the pandemic (Supplementary Fig. 13). It is possible that the viral variant associated with the critical care surge in Australia was less virulent than the COVID-19 variants associated with the early surges. Another important implication of the later timing of ICU admissions in Australia is that more of the Australian patients may have been vaccinated, which would be expected to protect against death and severe disease. However, only a limited supply of vaccines became available in February 2021 and vaccination was initially restricted to healthcare workers and high risk persons, resulting in a relatively low vaccination rate during the study period (ft.com/covid-vaccine). A more relevant consideration is that the timing of the pandemic in Australia may have permitted clinicians an opportunity to acquire knowledge about treating severe COVID-19, based on the experience of countries with heavy early surges. The analysis is limited by the amount of missing data (Tables 1 and 2). Patients without outcomes were excluded, thus missing data is not a factor for the evaluation of mortality by ethnicity. By contrast, a substantial number of treatment fields were unanswered, which renders the validity of comparing treatments by ethnicity questionable. In logistic regression, American Indian or Alaska Native had the lowest odds of missing APACHE II scores (Supplementary Fig. 14A). The proportion of missing scores showed a wide variation across sites (Supplementary Fig. 14B). We report some results even with a high amount of missing data, because of the objective of identifying challenging areas for conducting international research on ethnicity and because the illness severity data, albeit limited, may help explain healthcare disparities. Because the European Union does not have a uniform system or a harmonized requirement for collecting information on race and ethnicity, case report forms from Spain and Italy did not have any ethnicity fields, leading to the exclusion of 10,065 patients (Fig. 1). This highlights the need for alternative or supplementary categories to race and ethnicity for international studies.

Interpretation of differences in severity scores by ethnicity must be made with caution because commonly used scoring algorithms, including SOFA and APACHE II, have been noted to have statistical bias in mortality predictions for Black and Hispanic persons [28–30]. Developing more accurate and equitable illness severity scores, particularly for scenarios of surge conditions, is an important area for future research. Because case report forms did not include information on socioeconomic status, a crucial mediating variable of some differences in healthcare observed between ethnicity groups, we were unable to determine how much this contributed to the associations observed between ethnicity, disease severity, and outcomes [31]. Including information on socioeconomic status along with information on ethnicity is an important area for future research. For countries with legal or cultural policies prohibiting collection of information on ethnicity, other indicators of inequality or minority status may be important to include in case report forms, such as migration background [32]. Future research on disparities in healthcare should also include efforts to increase the opportunity for minorities and vulnerable groups to self-define identity terms relating to race and ethnicity [32].

## Conclusions

Among 7,394 patients from 32 countries with ICU admission for severe COVID-19, disease severity and mortality varied significantly by ethnicity. In the US cohort, American Indian or Alaska Native patients required dialysis and mechanical ventilation frequently and suffered higher mortality than other ethnicity groups. In the Australian cohort, Aboriginal/First Nations persons had the highest organ failure scores. The South African cohort, which was predominantly non-White, consistently required mechanical ventilation and had a high mortality rate. A public health implication of the study is that identification of sites and ethnicity groups with high mortality may identify vulnerable communities in need of culturally sensitive interventions to promote vaccination and to reduce community transmission. Clinicians caring for vulnerable ethnicity groups with severe COVID-19 should consider outreach to minimize delays in accessing critical care and optimization of strategies to avoid the need for mechanical ventilation- e.g., proning and non-invasive ventilation. Ethnicity groups with longstanding healthcare disparities presented with high acuity from severe COVID-19, frequently needed mechanical ventilation, and had high mortality. Because there is no global system of race and ethnicity classification, researchers designing case report forms for international registry studies should consider including related information, such as socioeconomic status or migration background.

### Supplementary Information

**Additional file 1: Supplementary Figure 1.** Diagram of time-to-event models: Model 1 is for the competing risks of discharge (discharged alive) and death. Model 2 is for competing risks of discharged alive, death, and mechanical ventilation. **Supplementary Figure 2.** 2A: Distribution of ethnicities in the US cohort, using US Census categories. 2B: Distribution of ethnicities in the Australian cohort, using the original case report form categories. 2C: Distribution of ethnicities in the South African cohort, using South African government categories. Percentages are given in each category and patient counts are along the X-axis. **Supplementary Figure 3.** Comorbidities, US Cohort. **Supplementary Figure 4.** Apache II scores by ethnicity, US Cohort. The box extends from the first to the third quartile. The line in the box is the median score. The upper and lower whiskers extend from the box to the largest and smallest scores, no further than 1.5 inter-quartile ranges from the box. Scores beyond the end of the whiskers are outlying points and are plotted individually. **Supplementary Figure 5A.** Cumulative probability of mortality and discharged alive, US cohort. For the designated ethnicity, the red curve represents the cumulative probability of death, the blue curve the cumulative probability of discharged alive, with days along the x-axis. **Supplementary Figure 5B.** Cumulative probability of mortality and discharged alive, Australian cohort. For the designated ethnicity, the red curve represents the cumulative probability of death, the blue curve the cumulative probability of discharged alive, with days along the x-axis. **Supplementary Figure 5C.** Cumulative probability of mortality and discharged alive, South African cohort. For the designated ethnicity, the red curve represents the cumulative probability of death, the blue curve the cumulative probability of discharged alive, with days along the x-axis. **Supplementary Figure 6A.** Hazard of death (left panel) and discharged alive (right panel) by ethnicity, US cohort, after adjustment by age and site. The blue dot is the hazard ratio for death (left panel) and discharged alive (right panel) and the blue whiskers are the 95% credible intervals. **Supplementary Figure 6B.** Hazard of death (left panel) and discharged alive (right panel) by ethnicity, US cohort, after adjustment only for age. The blue dot is the hazard ratio for death (left panel) and discharged alive (right panel) and the blue whiskers are the 95% credible intervals. **Supplementary Figure 6C.** Hazard of death (left panel) and discharged alive (right panel) by ethnicity, US cohort, after adjustment only for site. The blue dot is the hazard ratio for death (left panel) and discharged alive (right panel) and the blue whiskers are the 95% credible intervals. **Supplementary Figure 7.** Sensitivity analysis, allowing effect of ethnicity to vary by site using a site-specific effect for ethnicity in a random effects model. The hazard ratio for death for each site, compared to the average across all sites, is the central dot, with sites arranged vertically. The whiskers are 95% credible intervals. Sites with statistically significant increased or decreased hazard ratios for death, compared to the overall average for all sites (for the specified ethnicity), are in orange. **Supplementary Figure 8.** Comorbidities by ethnicity, Australian cohort. Designations are the same format as for Supplementary figure 2. **Supplementary Figure 9.** Sequential Organ Failure Assessment Score by Ethnicity, Australian Cohort: The box extends from the first to the third quartile. The line in the box is the median score. The upper and lower whiskers extend from the box to the largest and smallest scores, no further than 1.5 inter-quartile ranges from the box. Scores beyond the end of the whiskers are outlying points and are plotted individually. **Supplementary Figure 10A.** Hazard of discharged alive by ethnicity for each site, Australian cohort. Dots are hazard ratios and whiskers are 95% credible intervals. Sites are arranged vertically. Orange dots designate sites with hazard of discharged alive with statistically significant increased or decreased hazards of discharge, compared to the overall average across all sites for the specified ethnicity. **Supplementary Figure 10B.** Hazard of death by ethnicity for each site, Australian cohort. Dots are hazard ratios and whiskers are 95% credible intervals. Sites are arranged vertically. Orange dots designate sites with hazards with statistically significant increased or decreased hazards of death, compared to the overall average across all sites, for the specified ethnicity. **Supplementary Figure 11.** Sequential Organ Failure Assessment score by ethnicity, South African cohort. The box extends from the first to the third quartile. The line in the box is the median score. The upper and lower whiskers extend from the box to the largest and smallest scores, no further than 1.5 inter-quartile ranges from the box. Scores beyond the end of the whiskers are outlying points and are plotted individually. **Supplementary Figure 12.** A-C Cumulative probability of mechanical ventilation, US cohort (12A), Australian cohort (12B), South African cohort (12C). Y-axis- probability of mechanical ventilation, X-axis, days from first symptom. **Supplementary Figure 13.** The number of patients enrolled in COVID Critical (y-axis) based on date of admission (x-axis) for the U.S., South Africa, and Australia. **Supplementary Figure 14A.** Logistic regression for likelihood of missing APACHE II score by ethnicity. The reference group is the “Unanswered” ethnicity response category. Results below the reference line (less than an odds ratio of 1) are less likely to be missing; results above the reference line (odds ratio greater than 1) are more likely to be missing. **Supplementary Figure 14B.** Proportion of case reports missing APACHE II by Site, US Cohort. The site number is along the X-axis. The black dot indicates the proportion of cases from the site missing APACHE II scores.

**Additional file 2.**

## Data Availability

Please address inquiries regarding data availability to ECMOCARD@health.qld.gov.au Critical Care Research Group Prince Charles Hospital, Rode Road, Chermside, QLD, 4032 Australia. Questions can be initiated using the “Contact us” tab of the site: covid-critical.com Collaborators and affiliations are listed in the Acknowledgements section.

## Appendix 1

### Ethnicity categories of the case report forms

The REDCap case report form instrument includes a demographics section.

Item 26 is termed Ethnic group, with the instruction, “Check all that apply.”

Options are

1. 1 Arab
2. Black
3. East Asian
4. South Asian
5. West Asian
6. Latin American
7. White
8. Aborignial/First Nations
9. Other (Specify:_______)
10. N/A

## Appendix 2

### Responses to free text field to specify “other” ethnicity response

**Table:**

Specified Free Text Entry	n	Percent
South East Asian or Southeast Asian	100	18
Coloured	54	9.6
Asian	26	4.6
Hispanic or Hispanic/Latino	35	6.3
African	19	3.4
Brown	13	2.3
Javanese or Java	32	5.7
American Indian or Native American or Native American Indian	22	4
Greek	10	1.8
Not specified or Not Known or Unknown or Unknown/other	13	2.4
Balinese	**7**	1.2
Indonesian	8	1.5
Multiracial	6	1.1
Canadian	5	0.9
Macedonian	4	0.7
Southeast Asian (Indonesian)	10	1.8
Fiji	3	0.5
Iran/Iranian	5	0.9
Italian	4	0.7
Minahasa	3	0.5
Persian	4	0.7
Samoa or Samoan	6	1.1
Tonga or Tongan	4	0.7
Afghan	2	0.4
Asian Indian	2	0.4
East Europe/East European	5	1
Indian	3	0.6
Mexican	2	0.4
Mexican-American	3	0.6
Mixed Race	3	0.6
Multiracial/multicultural	2	0.4
Native Hawaiian or Native Hawaiian/Pacific Islander	6	1.2
Not Hispanic or Latino	4	0.8
Romanesa	2	0.4
Somali	2	0.4
Turkish	2	0.4
Albania or Albanian	2	0.4
Armenian	1	0.2
Asian- not specified	1	0.2
Asian Laotian	1	0.2
Asian Other	1	0.2
Australian	1	0.2
Bangladesh	1	0.2
Bosnian	1	0.2
Cook Islander	1	0.2
Czech Republic	1	0.2
East African	1	0.2
Egyptian	1	0.2
European - Iranian	1	0.2
Fiji / Hinduism	2	0.4
Georgia	1	0.2
Greek Orthodox	1	0.2
Gypsy (sic)	2	0.4
Hungarian	1	0.2
Lebanese/Arabic	1	0.2
Lebanon	1	0.2
Mediterranean	1	0.2
Mixed Race / Cape Malay	1	0.2
Native	1	0.2
Nepali	1	0.2
Non-hispanic, non-latino Asian	1	0.2
Other	1	0.2
Pakistan or Pakistani	2	0.4
Philippines or Philipino (sic) or South East Asian - Philippines	3	0.6
Russian	1	0.2
Slovakian	1	0.2
Spanish	1	0.2

## Appendix 3

### South African demography and healthcare system relevant to COVID critical ethnicity analysis for South Africa

The South African data, although low in numbers, highlighted a number of important findings as well as interpretation difficulties in assessing race and ethnicity within a global consortium.

First, the categorization of race used for the South Africa COVID Critical analysis was more consistent with the national government classification system (superseding the ISARIC CRF classification). This system reflects a legacy of apartheid. Race was unlikely to be self identified by the patient themselves as 100% of patients were mechanically ventilated and 90% paralyzed on enrollment and there was a 70% mortality rate.

In 28% of patients “Other” racial classification was selected from the case report form and free text was entered to designate the patients as “Coloured”. In some countries, interpretation of this term would result in patients being classified as “Black”. In South Africa the Coloured community is a genetically highly admixed but discrete ethnic group comprised of indigenous Khoi and San (Khoisan), tribal Bantu-speaking populations, European settlers, and descendants of persons who arrived on slave ships from Java, India, Mozambique and Madagascar [24]. In South Africa this group makes up 9% of the national population. The largest race and ethnicity group in our population was “Black,” accounting for 50% of patients. This compares to 79.2% of the national population who were classified as black in the most recent national census.

There would therefore appear to be a disproportionate access to intensive care resources for different racial groups in comparison with the national census data. It is important to realize, however, that the regional distribution of population groups is highly varied in South Africa and that healthcare outcomes as assessed by average life expectancy differ markedly between these regions as do average annual household income levels.

National census data and census data from 4 provinces within South Africa with population groupings, average life expectancy, annual average household income and medical insurance per population group [33, 34]

**Table:**

	SA Analysis of Covid Critical Outcomes	SA national census data 2011	Western Cape census data	Kwa-Zulu Natal census data	Gauteng census data	Eastern Cape census data	Private medical insurance (national)
Total population	-	51,770,560	5,822,734	10,267,300	12,272,263	6,562,053	16.9%
Black	50%	79.2%	32.9%	86.8%	77.4%	86.3%	10.1%
Coloured	28%	8.9%	48.8%	1.4%	3.5%	8.3%	20.2%
White	< 1%	8.9%%	15.7%	4.2%	15.6%	4.7%	72.4%
Indian/Asian	2%	2.5%	1%	7.4%	2.9%	0.4%	48.9%
Missing	16%	-	-	-	-	-	-
Other (Not coloured)	2%	1.6%	1.6%	0.3%	0.3%	0.3%	-
Average life expectancy (female and male)	-	-	71.1 and 65.7	63.7 and 57.1	69.2 and 63.8	67.1 and 59.6	-
Average annual household income	-	R 103 204 $6 376	R143 460 $8 877	R83 053 $5140	R156 243 $9 669	R64 539 $3994	

An assessment of the possible impact of race and ethnicity on Covid-19 outcomes in South Africa also should also include consideration of a markedly discrepant two-tiered healthcare system. Access to private medical insurance differs dramatically between population groups in South Africa with 72.4% of the white population and only 10.1% of the Black population able to access private healthcare insurance. There is a 10-fold difference in per capita spending on healthcare between the public ($140 annually) and private ($ 1400 annually) healthcare sectors in South Africa [22].

In a country with years of formally institutionalized discrimination, access to healthcare has a major impact in the racial determination of outcomes from disease that we were not able to analyze due to the multiple confounders. Disparities in healthcare insurance and healthcare delivery are targeted for redress with a national health insurance program [21]. The poor outcomes in the South African cohort is a reflection that patients were all invasively mechanically ventilated (as per triage guidelines for scarce ICU resources) with limited ECMO provision [35]. The male to female ratio was 50/50 for both the Coloured and Black population groups, which matched with the overall global analysis of Black patients but differed from gender distribution of other race and ethnicity groups, where male sex predominated.

## Appendix 4

### Framework for including race and ethnicity in case report forms for registry studies

Recommendation 1: Please use race and ethnicity categories specified in terms of your national census.

Recommendation 2: Please ask the patient, or a surrogate representing the patient, when the patient is unable to communicate, to self-designate the ethnicity description best fitting his or her identity.

Recommendation 3: If the patient (or family/surrogate) elects to not participate, please ask whether the reason is one of the following:

1. Concern regarding discrimination (unfair treatment) based on race and ethnicity designation (Yes/No/Prefer not to answer)
2. Categories available are not adequate

If the patient has an ethnicity identifier not included in the national census, and elects to participate in providing another designation, please specify:

Recommendation 4: Alternative, complementary, and related identifiers:

If the patient choses to disclose the information and gathering the information is allowed, please designate whether the patient endorses being included in the following designations:

A) Refugee/migrant worker/asylum seeker? (Yes/no/not applicable)
B) Uninsured/self-pay status, or seeking government or public assistance for medical care? (Yes/no/not applicable)
C) Employment status (Prior to illness and need for medical care)
