# Supplementary material for: Race and ethnicity in the COVID-19 Critical Care Consortium: demographics, treatments, and outcomes, an international observational registry study
Source: Int J Equity Health. 2023 Dec 12;22:260. doi: 10.1186/s12939-023-02051-w (PMC10717789; doi:10.1186/s12939-023-02051-w)
Supplement: Supplementary file 1 — Additional file 1: Supplementary Figure 1. Diagram of time-to-event models: Model 1 is for the competing risks of discharge (discharged alive) and death. Model 2 is for competing risks of discharged alive, death, and mechanical ventilation. Supplementary Figure 2. 2A: Distribution of ethnicities in the US cohort, using US Census categories. 2B: Distribution of ethnicities in the Australian cohort, using the original case report form categories. 2C: Distribution of ethnicities in the South African cohort, using South African government categories. Percentages are given in each category and patient counts are along the X-axis. Supplementary Figure 3. Comorbidities, US Cohort. Supplementary Figure 4. Apache II scores by ethnicity, US Cohort. The box extends from the first to the third quartile. The line in the box is the median score. The upper and lower whiskers extend from the box to the largest and smallest scores, no further than 1.5 inter-quartile ranges from the box. Scores beyond the end of the whiskers are outlying points and are plotted individually. Supplementary Figure 5A. Cumulative probability of mortality and discharged alive, US cohort. For the designated ethnicity, the red curve represents the cumulative probability of death, the blue curve the cumulative probability of discharged alive, with days along the x-axis. Supplementary Figure 5B. Cumulative probability of mortality and discharged alive, Australian cohort. For the designated ethnicity, the red curve represents the cumulative probability of death, the blue curve the cumulative probability of discharged alive, with days along the x-axis. Supplementary Figure 5C. Cumulative probability of mortality and discharged alive, South African cohort. For the designated ethnicity, the red curve represents the cumulative probability of death, the blue curve the cumulative probability of discharged alive, with days along the x-axis. Supplementary Figure 6A. Hazard of death (left panel) and discharged alive (r [file 12939_2023_2051_MOESM1_ESM.docx]

**Supplementary Material**

**Supplementary Figure 1**: Diagram of time-to-event models: Model 1 is for the competing risks of discharged alive and death. Model 2 is for competing risks of discharged alive, death, and mechanical ventilation.

**Supplementary Figure 2**: A: Distribution of ethnicities in the US cohort, using US Census categories. B: Distribution of ethnicities in the Australian cohort, using the original case report form categories. C: Distribution of ethnicities in the South African cohort, using South African government categories. Percentages are given in each category and patient counts are along the X-axis.

**Supplementary Figure 3** : Comorbidities, US Cohort. For each comorbidity, the red bar is the percentage of the designated ethnicity group with the condition, the blue bar is the percentage without the condition, and gray bars are the percentage of patients with no response for the given ethnicity and comorbidity. Comorbidity definitions are specified in the ISARIC COVID-19 Case report form, available at isaric.org: Chronic Kidney Disease, “any of estimated glomerular filtration rate <60 ml/min/1.73m2, history of kidney transplantation”; Diabetes, “Type 1 or Type 2 diabetes mellitus requiring oral or subcutaneous treatment”; Hypertension, “Elevated arterial blood pressure diagnosed clinically, >140mm Hg systolic or >90 diastolic”; Liver disease is either mild, “This is defined as cirrhosis without portal hypertension or chronic hepatitis”; or moderate or severe, “This is defined as cirrhosis with portal hypertension, with or without bleeding or a history of variceal bleeding”; Malnutrition, “any clinically defined deficiency in intake, either of total energy or of specific nutrients that led to a dietetic intervention or referral prior to the onset of COVID-19 symptoms”; Obesity, “BMI 30 or more”; Smoking, “Smoking at least one cigarette, cigar, pipe or equivalent per day before the onset of the current illness.”

**Supplementary Figure 4** : Apache II scores by ethnicity, US Cohort. The box extends from the first to the third quartile. The line in the box is the median score. The upper and lower whiskers extend from the box to the largest and smallest scores, no further than 1.5 inter-quartile ranges from the box. Scores beyond the end of the whiskers are outlying points and are plotted individually.

**Supplementary Figure 5A**: Cumulative probability of mortality and discharged alive, US cohort. For the designated ethnicity, the red curve represents the cumulative probability of death, the blue curve the cumulative probability of discharged alive, with days along the x-axis.

**Supplementary Figure 5B**: Cumulative probability of mortality and discharged alive, Australian cohort. For the designated ethnicity, the red curve represents the cumulative probability of death, the blue curve the cumulative probability of discharged alive, with days along the x-axis.

**Supplemental Figure 5C**: Cumulative probability of mortality and discharged alive, South African cohort. For the designated ethnicity, the red curve represents the cumulative probability of death, the blue curve the cumulative probability of discharged alive, with days along the x-axis.

**Supplementary Figure 6A**: Hazard of death (left panel) and discharged alive (right panel) by ethnicity, US cohort, after adjustment by age and site. The blue dot is the hazard ratio for death (left panel) and discharged alive (right panel) and the blue whiskers are the 95% credible intervals.

**Supplementary Figure 6B**: Hazard of death (left panel) and discharged alive (right panel) by ethnicity, US chort, after adjustment only for age. The blue dot is the hazard ratio for death (left panel) and discharged alive (right panel) and the blue whiskers are the 95% credible intervals.

**Supplementary Figure 6C**: Hazard of death (left panel) and discharged alive (right panel) by ethnicity, US chort, after adjustment only for site. The blue dot is the hazard ratio for death (left panel) and discharged alive (right panel) and the blue whiskers are the 95% credible intervals.

**Supplementary Figure 7**: Sensitivity analysis, allowing effect of ethnicity to vary by site using a site-specific effect for ethnicity in a random effects model. The hazard ratio for death for each ethnicity is the central dot, with sites arranged vertically. The whiskers are 95% credible intervals. Sites with statistically significant increased or decreased hazard ratios for death, compared to the overall average for all sites (for the specified ethnicity), are in orange.

**Supplementary Figure 8**: Comorbidities by ethnicity, Australian cohort. Designations are the same format as for Supplementary figure 3.

**Supplementary Figure 9**: Sequential Organ Failure Assessment Score (SOFA Score) by Ethnicity, Australian Cohort: The box extends from the first to the third quartile. The line in the box is the median score. The upper and lower whiskers extend from the box to the largest and smallest scores, no further than 1.5 inter-quartile ranges from the box. Scores beyond the end of the whiskers are outlying points and are plotted individually.

**Supplementary Figure 10A**: Hazard of discharged alive by ethnicity for each site, Australian cohort. Dots are hazard ratios and whiskers are 95% credible intervals. Sites are arranged vertically. Orange dots designate sites with hazard of discharged alive with statistically significant increased or decreased hazards of discharge, compared to the overall average across all sites for the specified ethnicity.

**Supplementary Figure 10B**: Hazard of death by ethnicity for each site, Australian cohort. Dots are hazard ratios and whiskers are 95% credible intervals. Sites are arranged vertically. Orange dots designate sites with hazards with statistically significant increased or decreased hazards of death, compared to the overall average across all sites, for the specified ethnicity.

**Supplementary Figure 11**: Sequential Organ Failure Assessment score by ethnicity, South African cohort. The box extends from the first to the third quartile. The line in the box is the median score. The upper and lower whiskers extend from the box to the largest and smallest scores, no further than 1.5 inter-quartile ranges from the box. Scores beyond the end of the whiskers are outlying points and are plotted individually.

**Supplementary Figure 12 A-C**: Cumulative probability (y-axis) of mechanical ventilation (red curve), death (black curve), or discharged alive (gray curve), US cohort (14A), Australian cohort (14B), and South African cohort (14C), with days from first symptom along x-axis.

**Supplemental Figure 13**: The number of patients enrolled in COVID Critical (y-axis) based on date of admission (x-axis) for the U.S., South Africa, and Australia

**Supplementary Figure 14 A**: Logistic regression for likelihood of missing APACHE II score by ethnicity. The reference group is the “Unanswered” ethnicity response category. Results below the reference line (less than an odds ratio of 1) are less likely to be missing; results above the reference line (odds ratio greater than 1) are more likely to be missing.

**Supplementary Figure 14 B:** Proportion of case reports missing APACHE II by Site, US Cohort. The site number is along the X-axis. The black dot indicates the proportion of cases from the site missing APACHEII Scores.

.

**Supplementary Figure 1: Diagram of time-to-event models**

Model 1: Time-to-event model for competing risks of discharged alive and death.

Model 2: Time-to-event model for competing risks of survival to discharge, death, and mechanical ventilation.


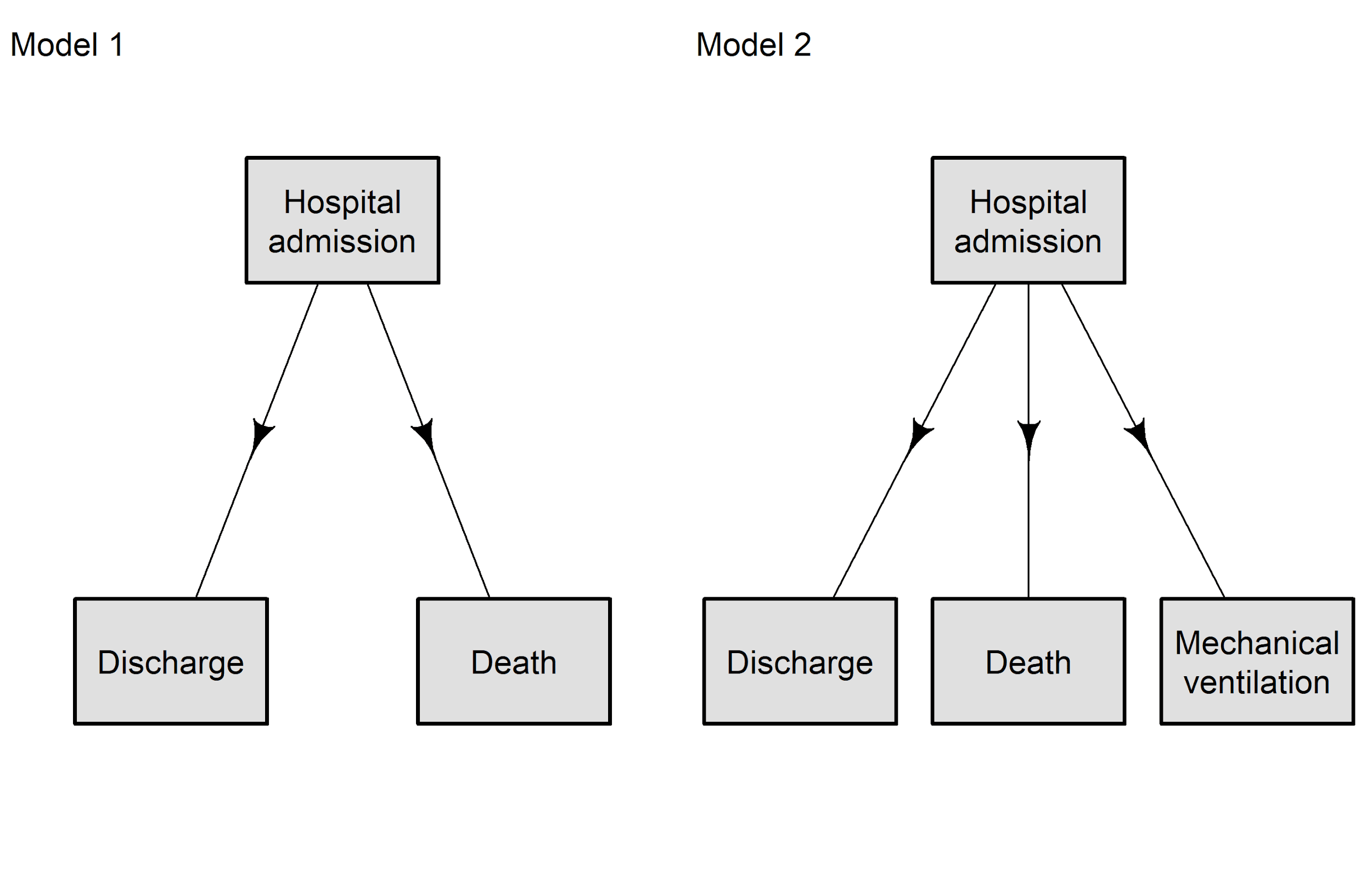


**Supplementary Figure 2A: Ethnicity for COVID Critical, US Cohort, B: Ethnicity for Australian Cohort, C: Ethnicity for South African Cohort**

**
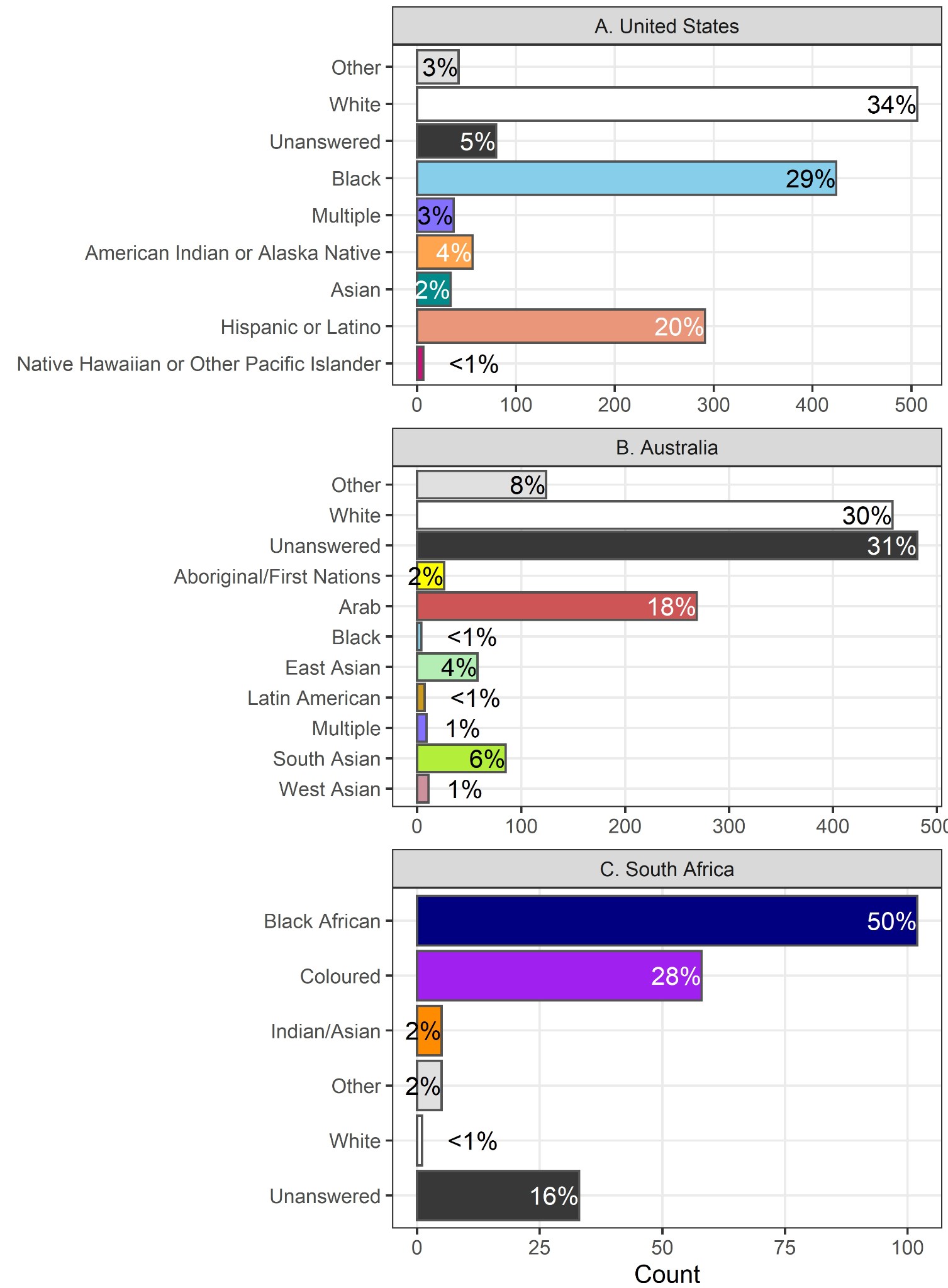
**

**Supplementary Figure 3, Comorbidities, US Cohort**

**
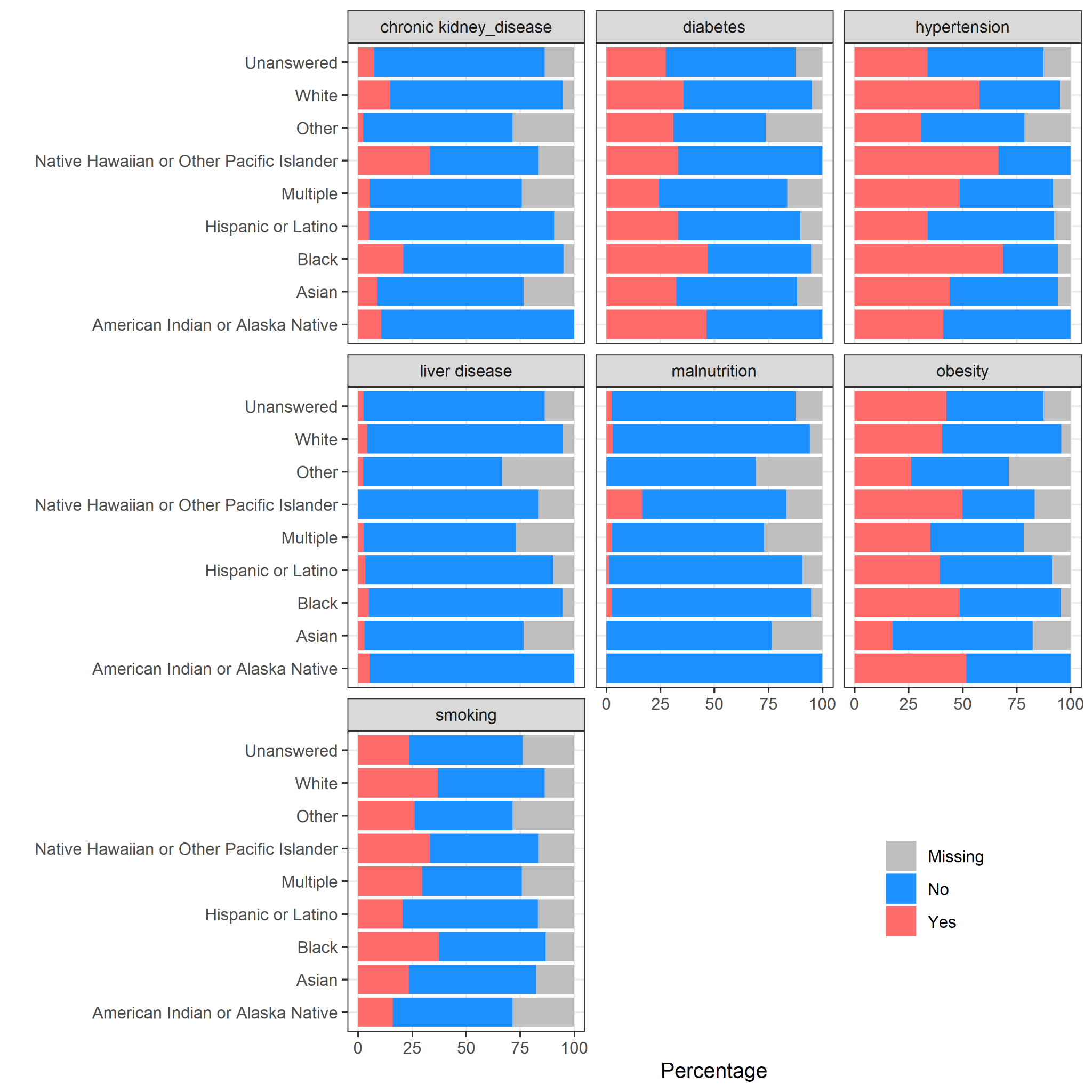
**

**Supplementary Figure 4, APACHE II, US Cohort**

**
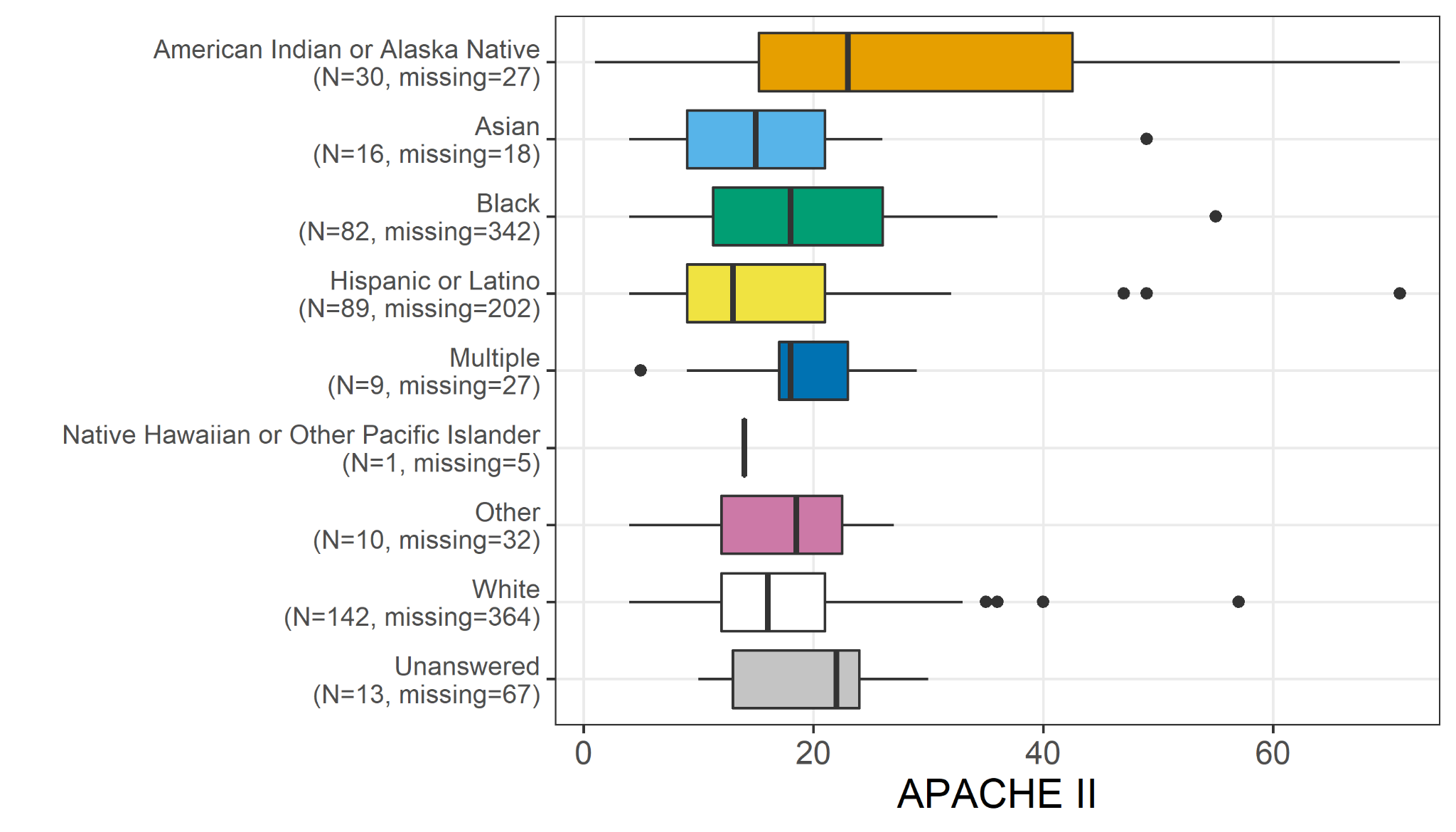
**

**Supplementary Figure 5A. Cumulative Mortality and discharged alive by Ethnicity, US Cohort**

**
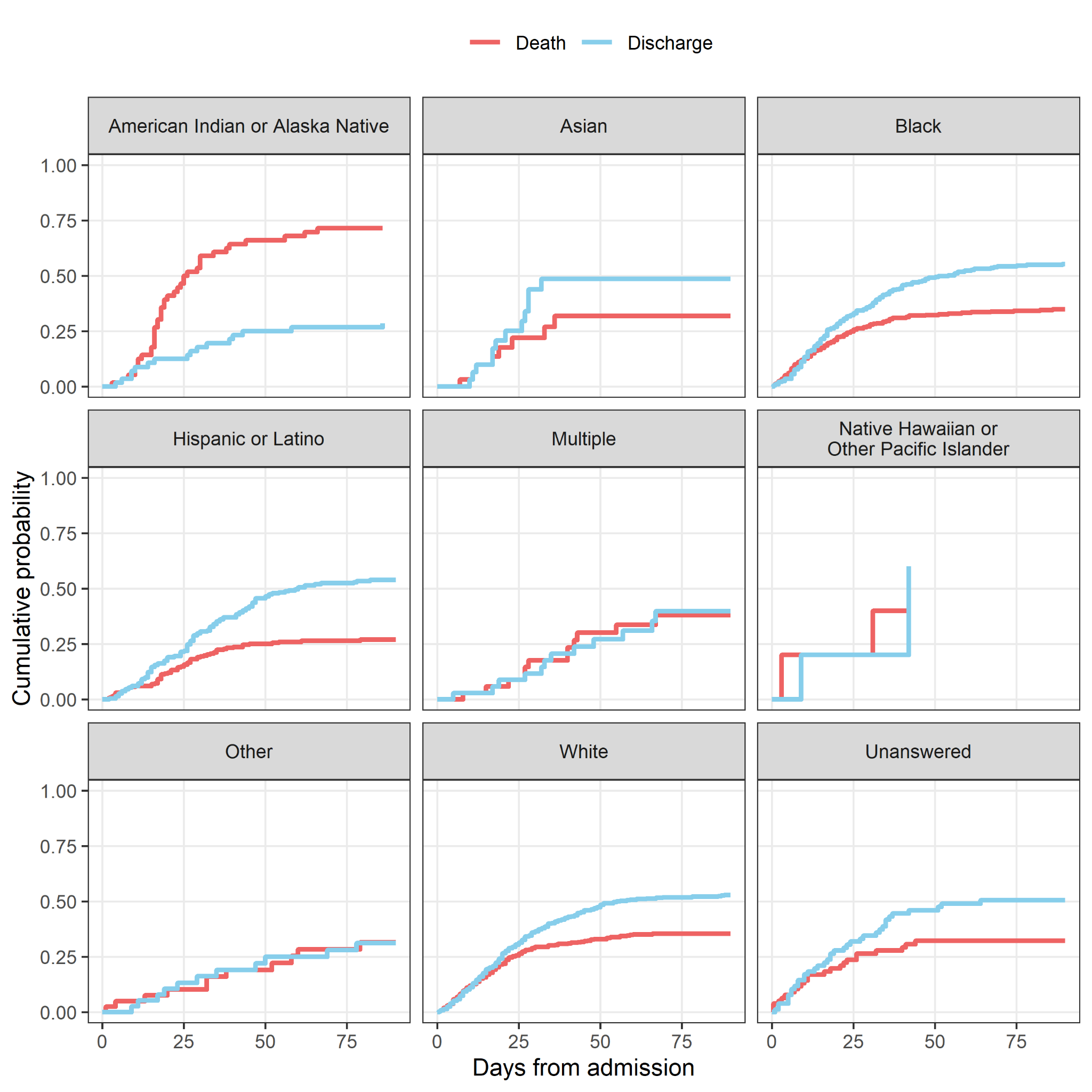
**

**Supplemental Figure 5B. Cumulative Mortality and discharged alive by Ethnicity, Australia Cohort**

**
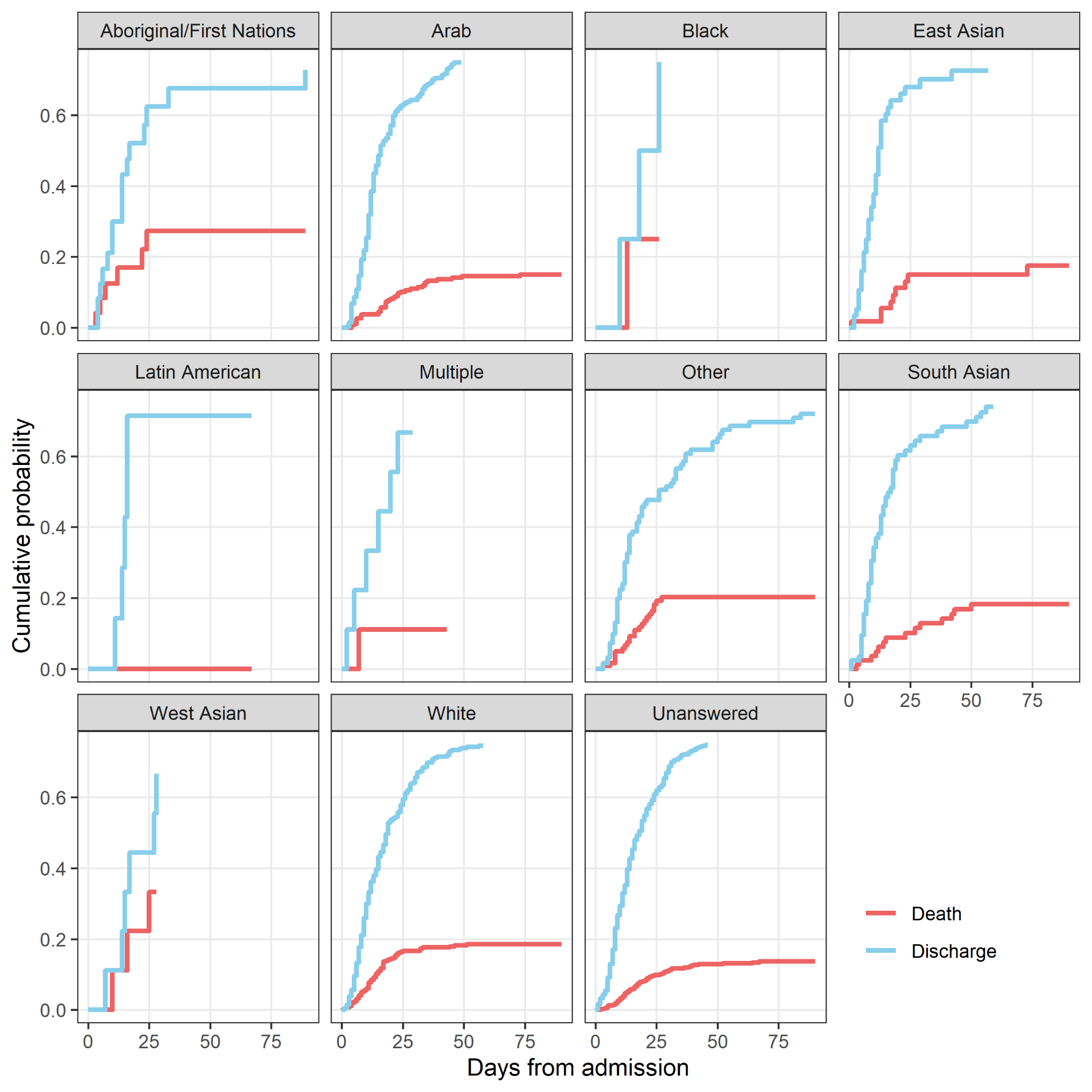
**

**Supplementary Figure 5C. Cumulative Mortality and discharged alive by Ethnicity, South Africa Cohort**

**
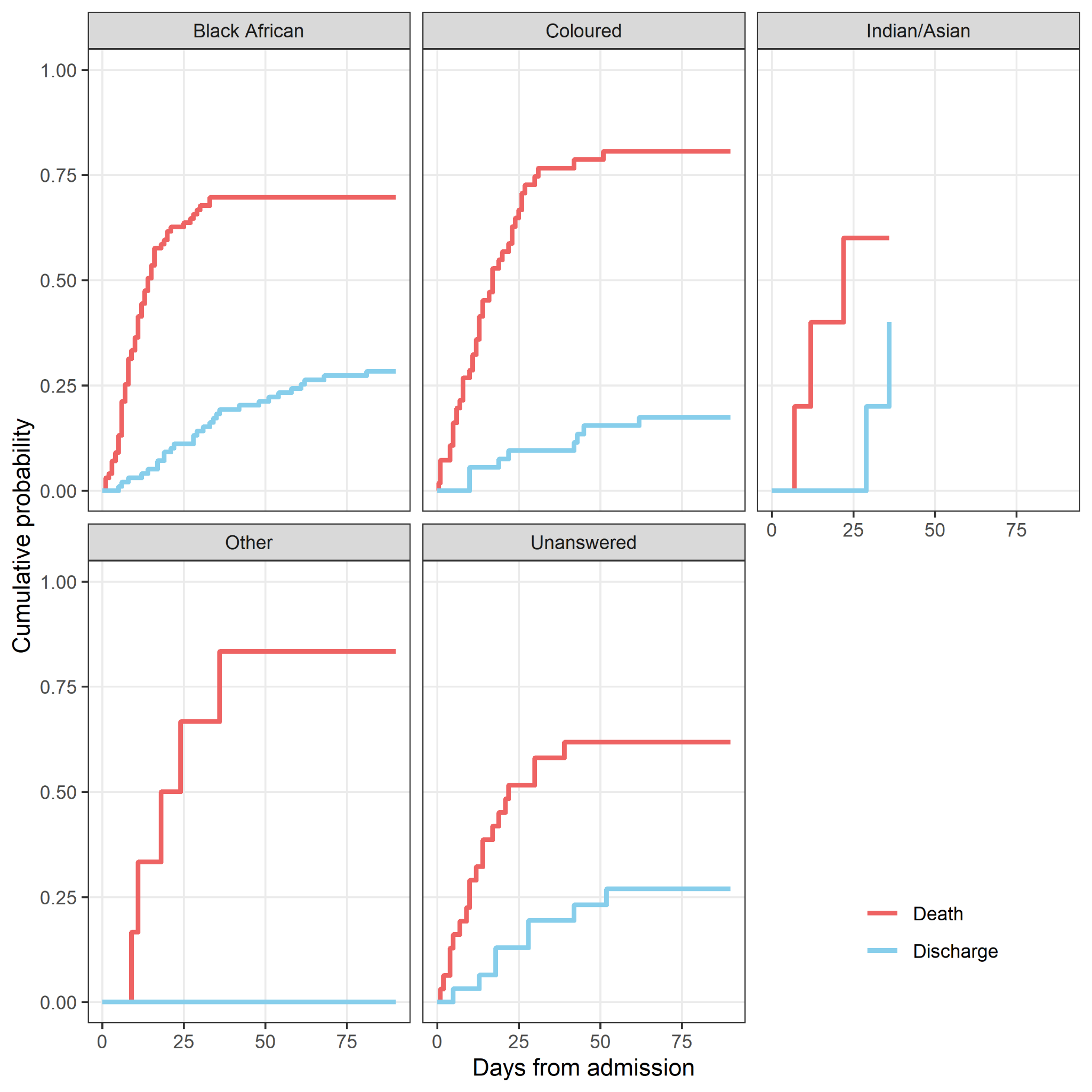
**

**Supplemental Figure 6A. In-hospital mortality, adjusted by site and age, US**

**
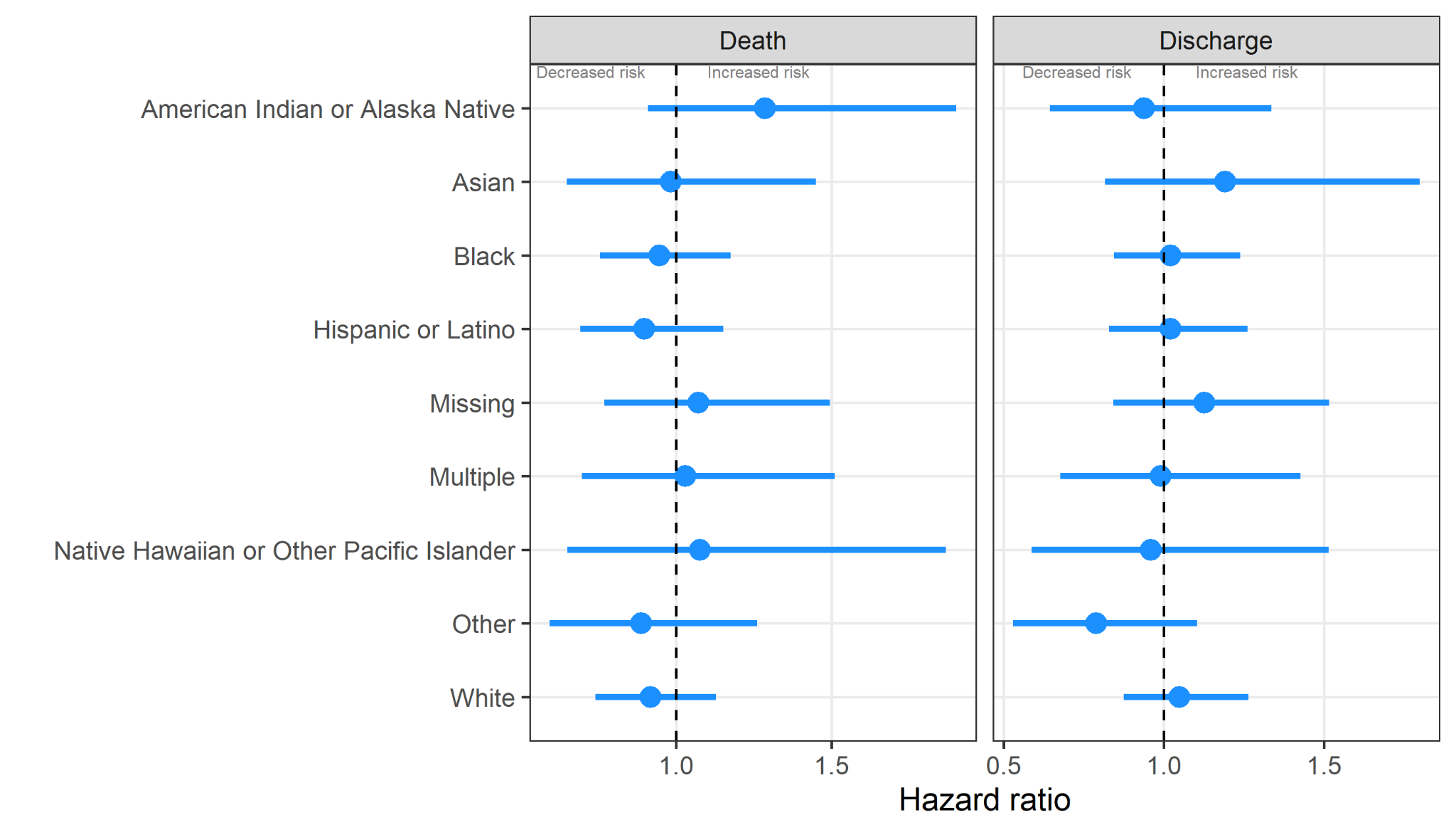
**

**Supplementary Figure 6B: Hazard of Death and discharged alive by Ethnicity, Adjusted by Age Only, US Cohort**


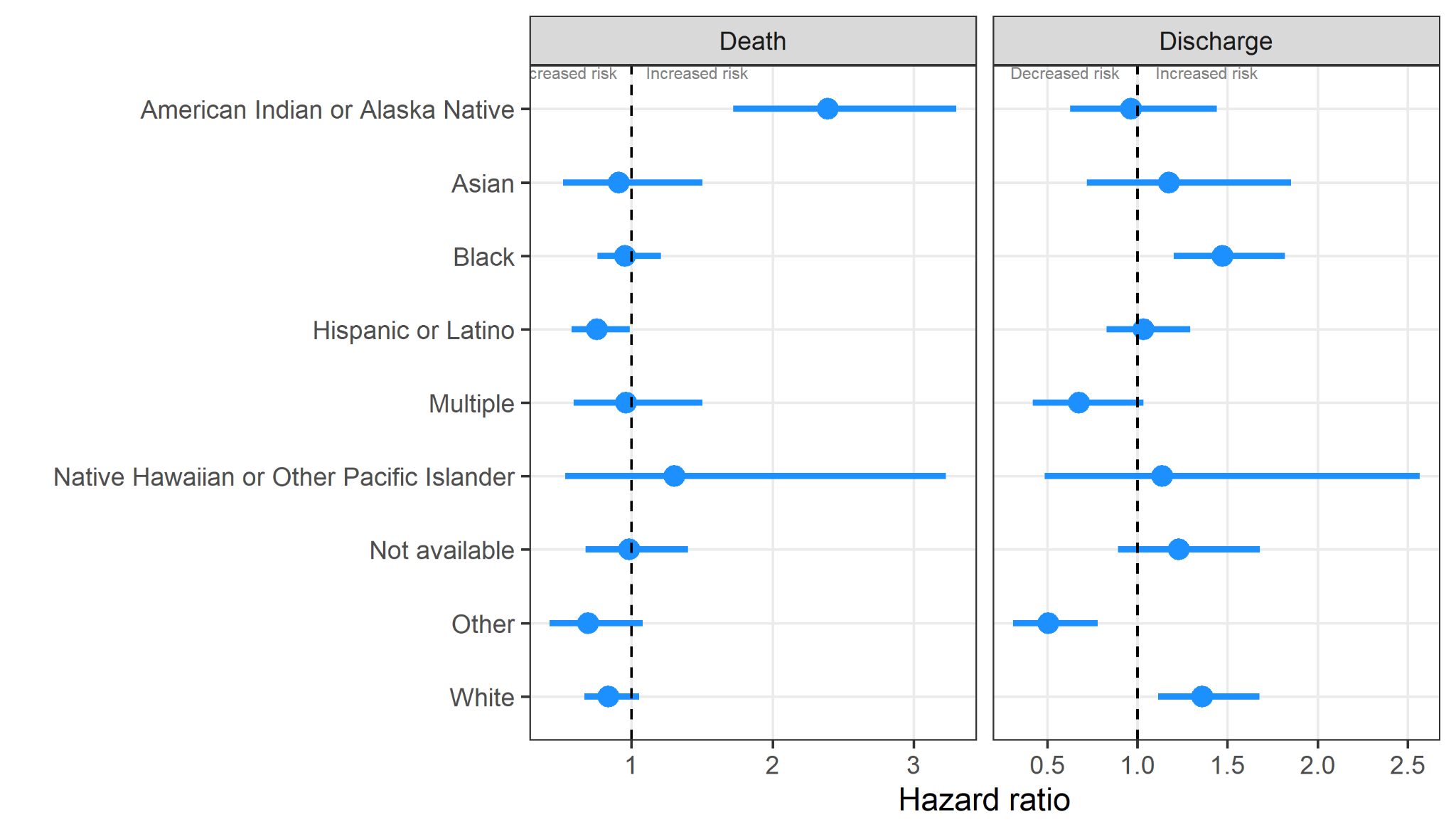


**Supplementary Figure 6C. Hazard of Death and discharged alive by Ethnicity, Adjusted Only for Site (US Cohort)**


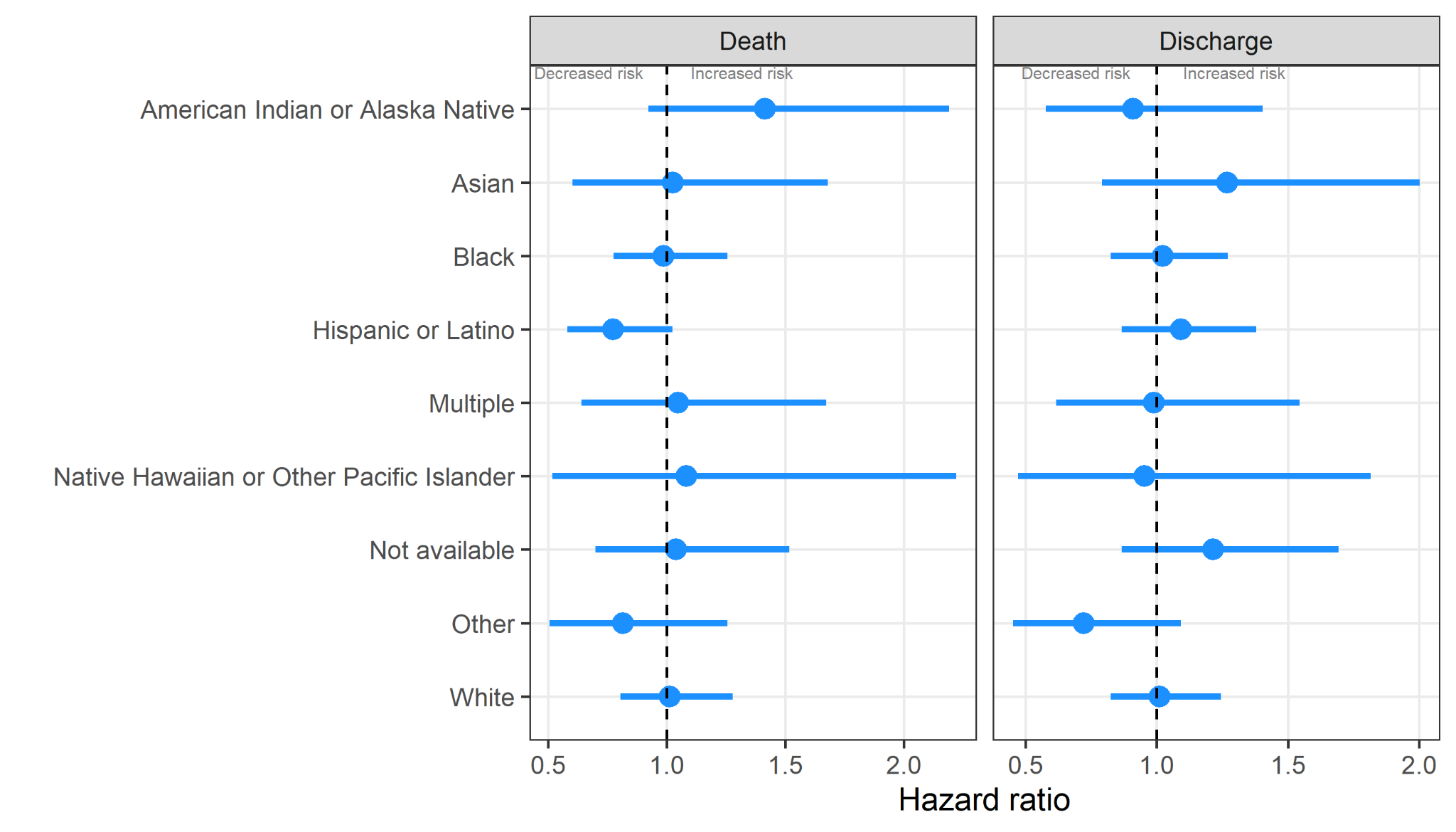


**Supplementary Figure 7 Variation in risk of death by ethnicity across US Sites, sites with statistically significant variation are in orange.**


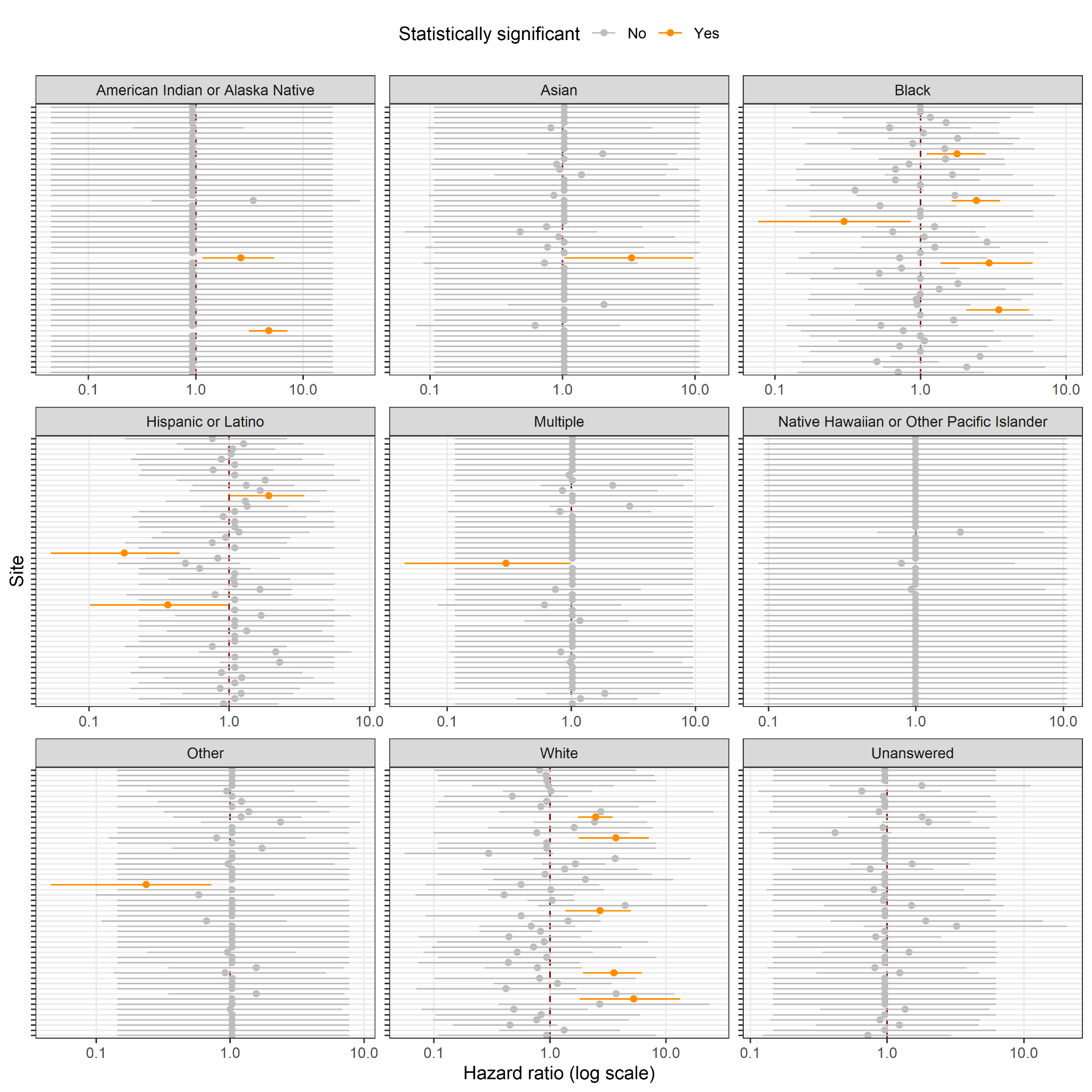


Supplementary Figure 8: Comorbidities by Ethnicity, Australian Cohort


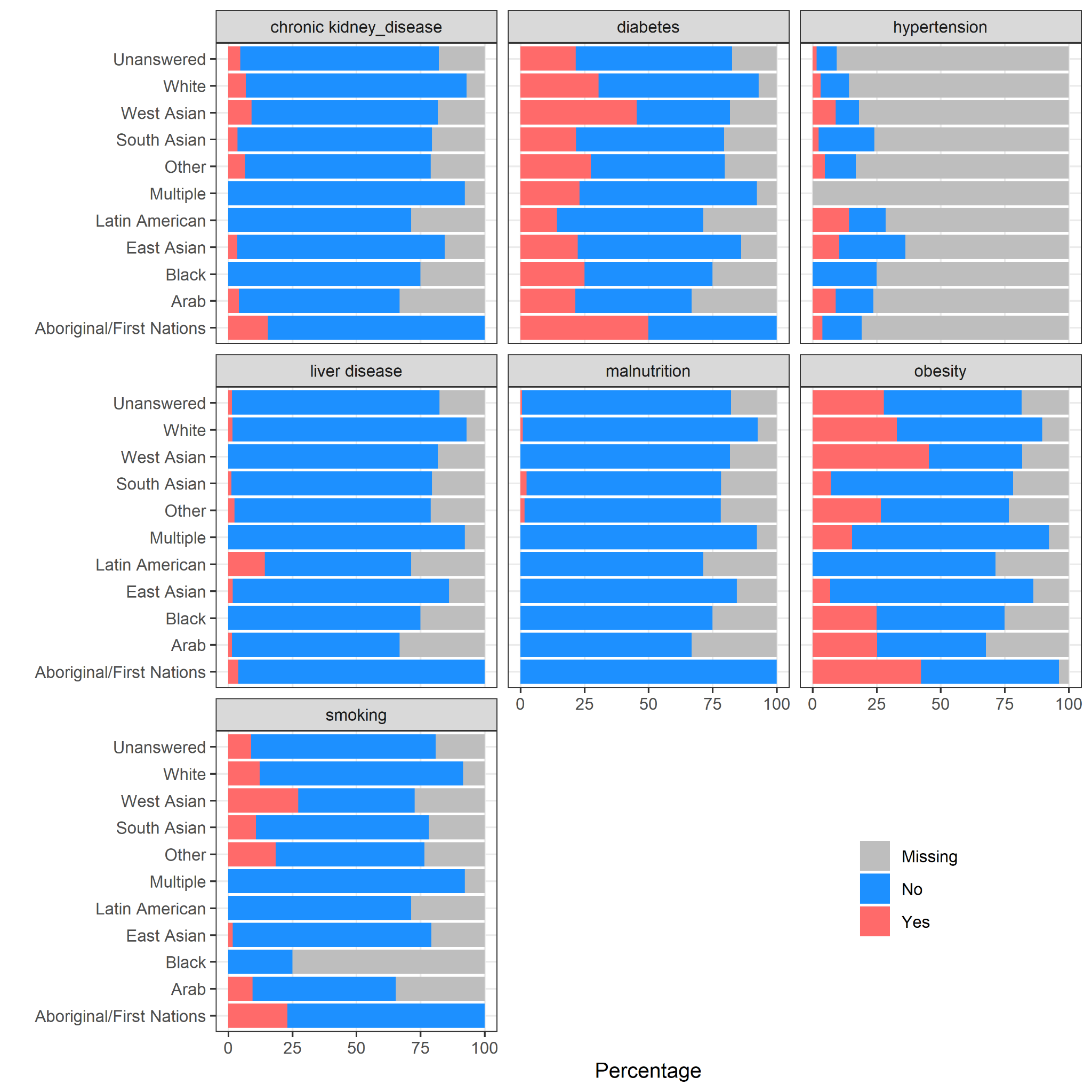


Supplementary Figure 9: SOFA Score by Ethnicity, Australian Cohort


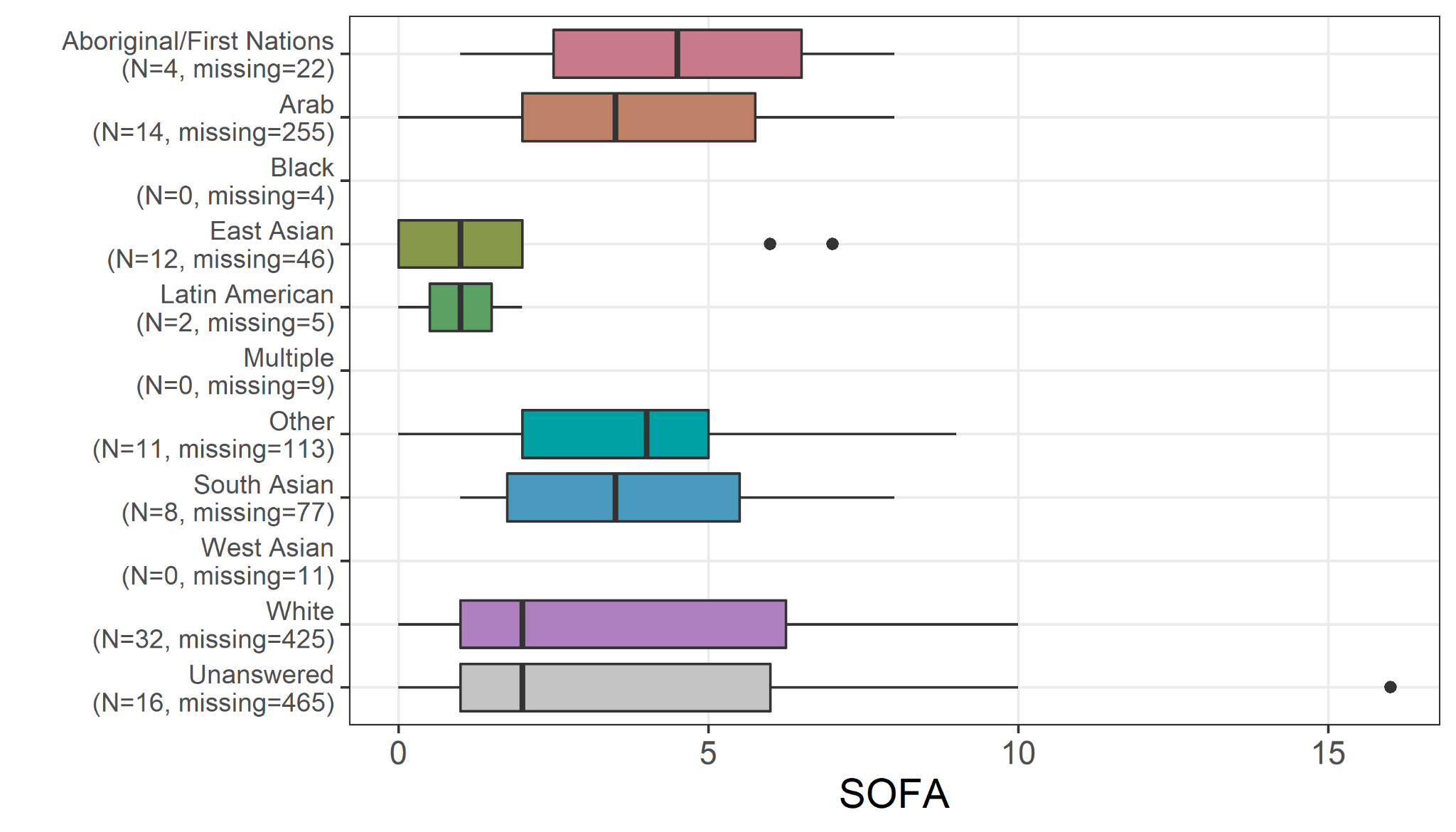


Supplementary Figure 10A: Variation of risk of discharged alive by ethnicity across Australian sites. Sites with significant variation for the specified ethnicity (with credible interval not including 1) are in orange.


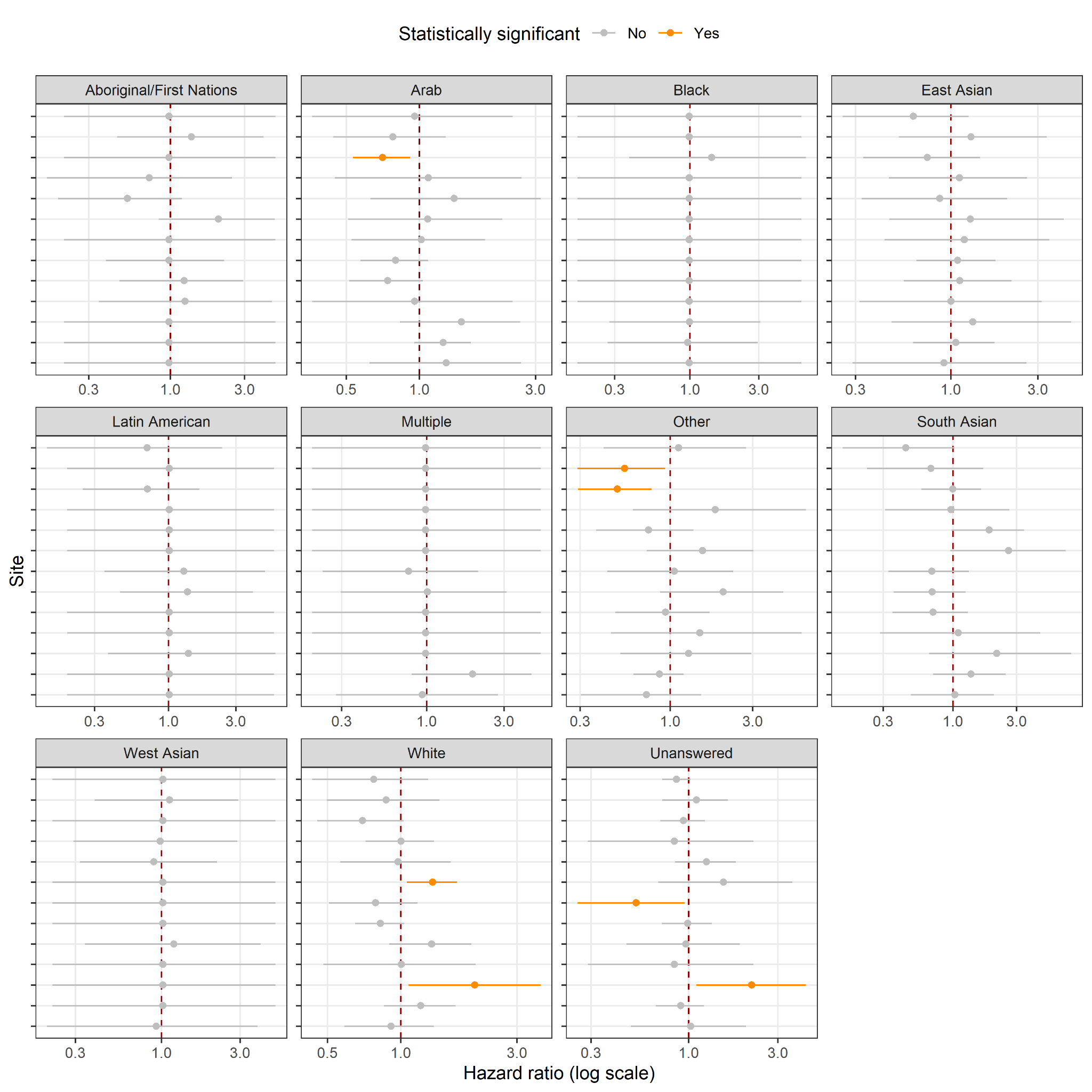


Supplementary Figure 10B: Variation of risk of death by ethnicity across Australian sites, sites with significant variation for the specified ethnicity (compared to the average across all sites) are in orange.
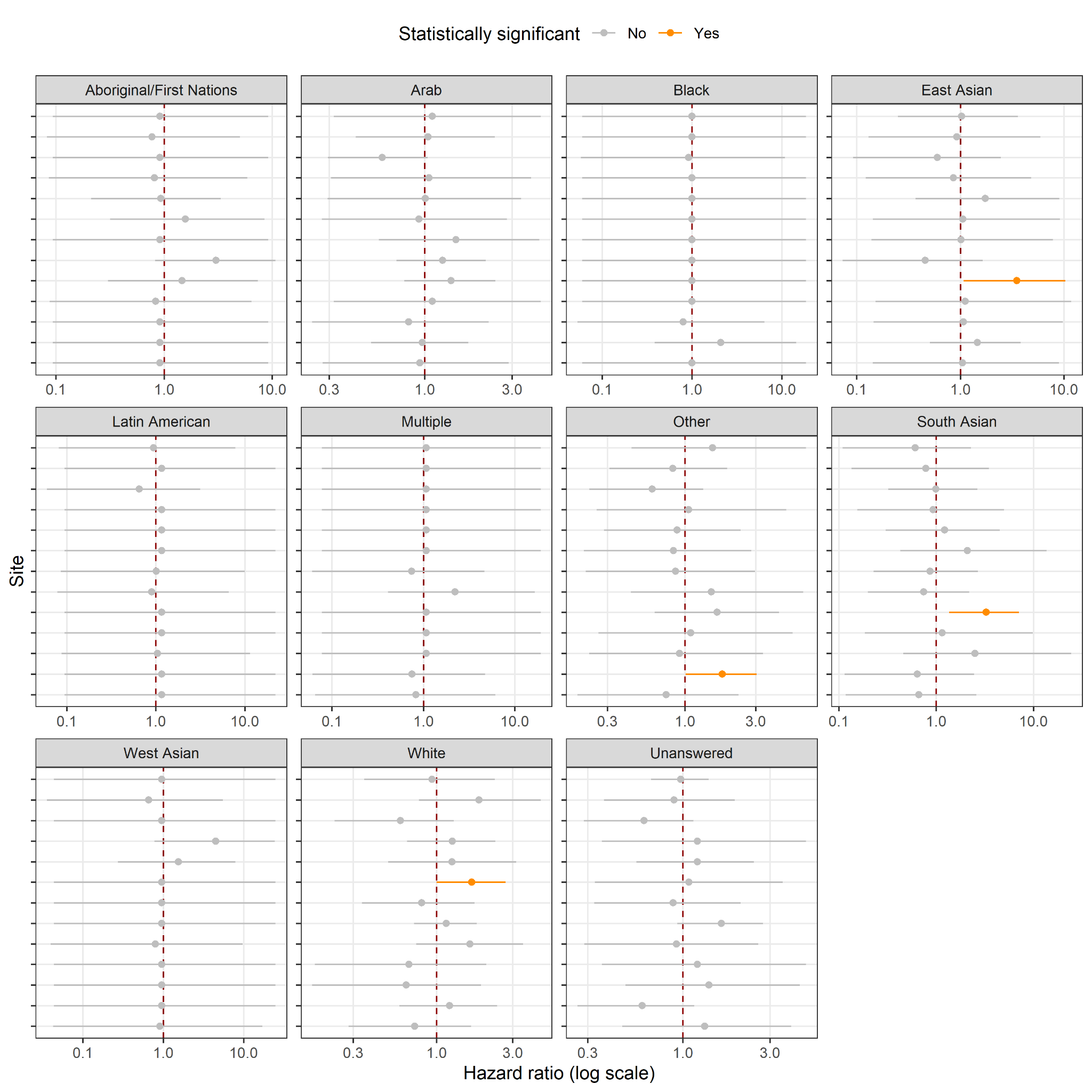


Supplementary Figure 11: SOFA scores by Ethnicity, South African Cohort


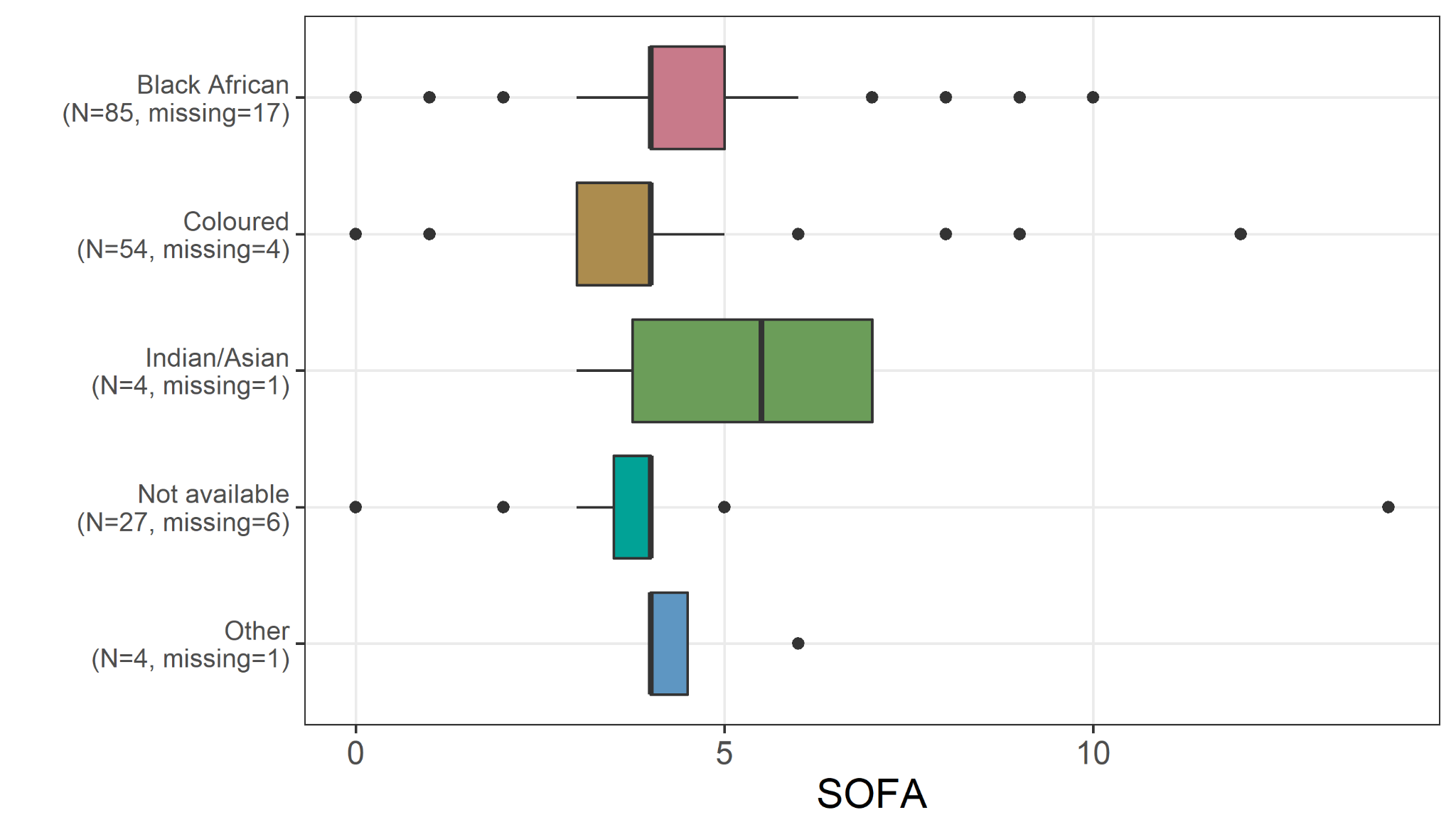


Supplementary Figure 12A: Cumulative probability of mechanical ventilation, US Cohort


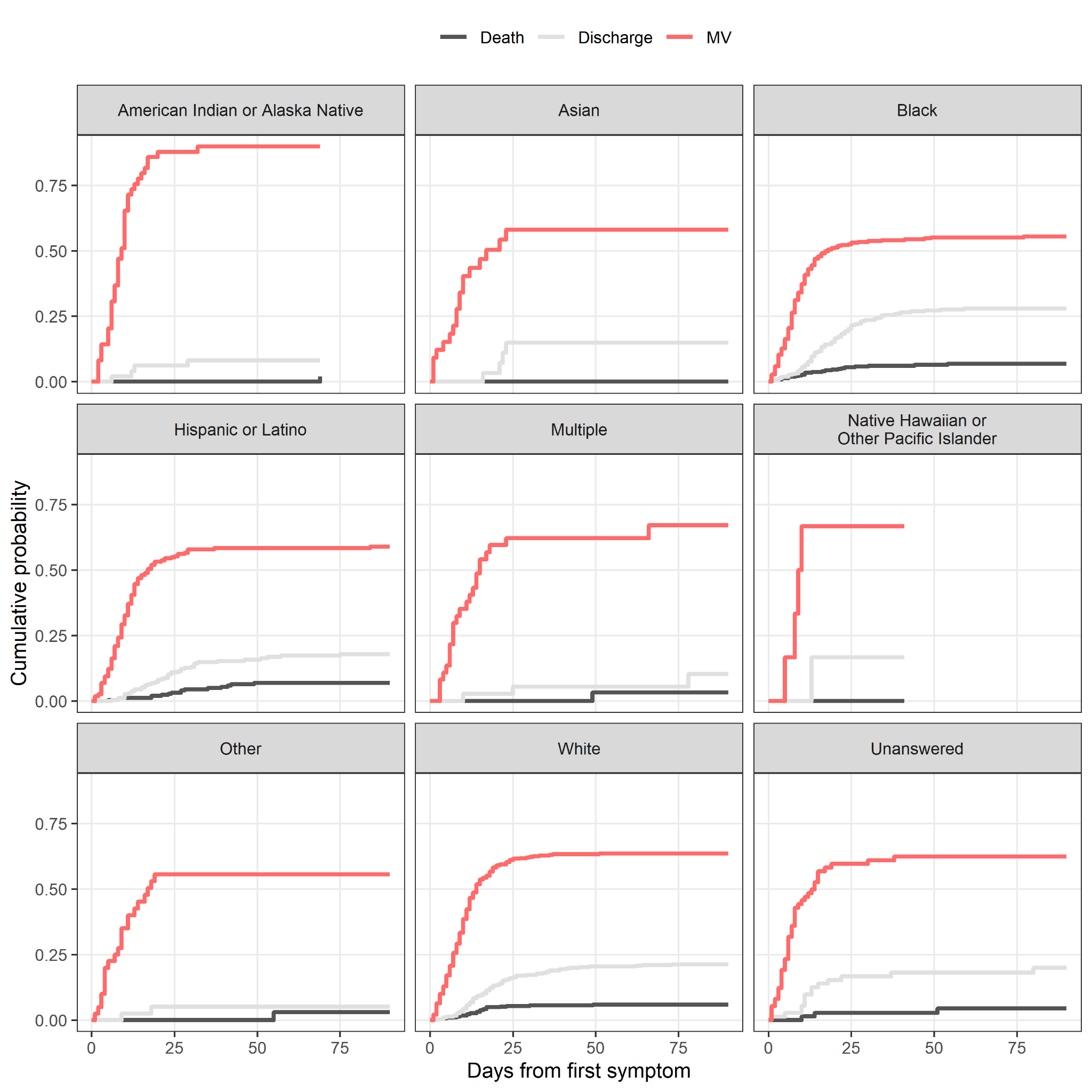


Supplementary Figure 12 B: Cumulative probability of mechanical ventilation, Australian Cohort


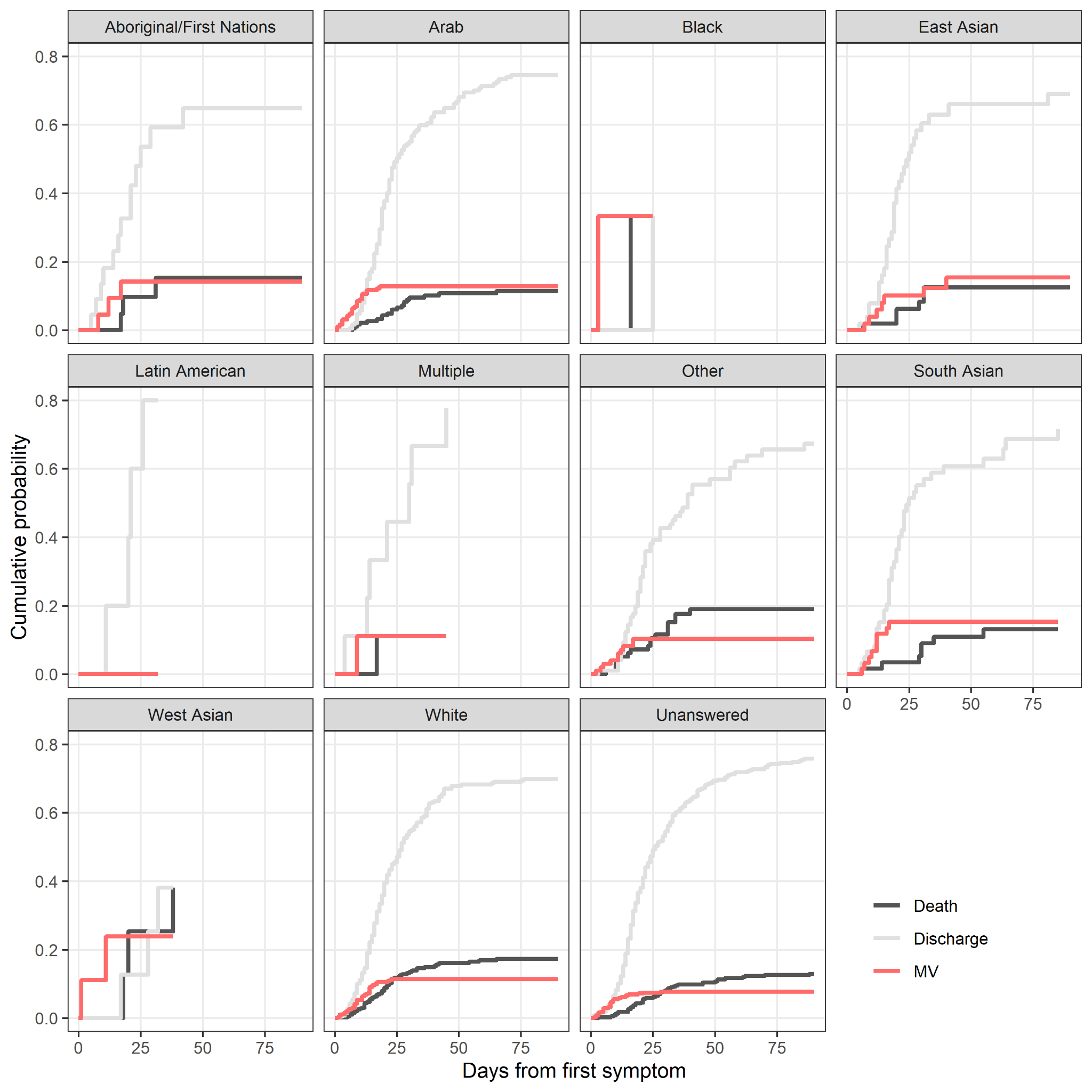


Supplementary Figure 12 C: Cumulative probability of invasive mechanical ventilation, South African Cohort


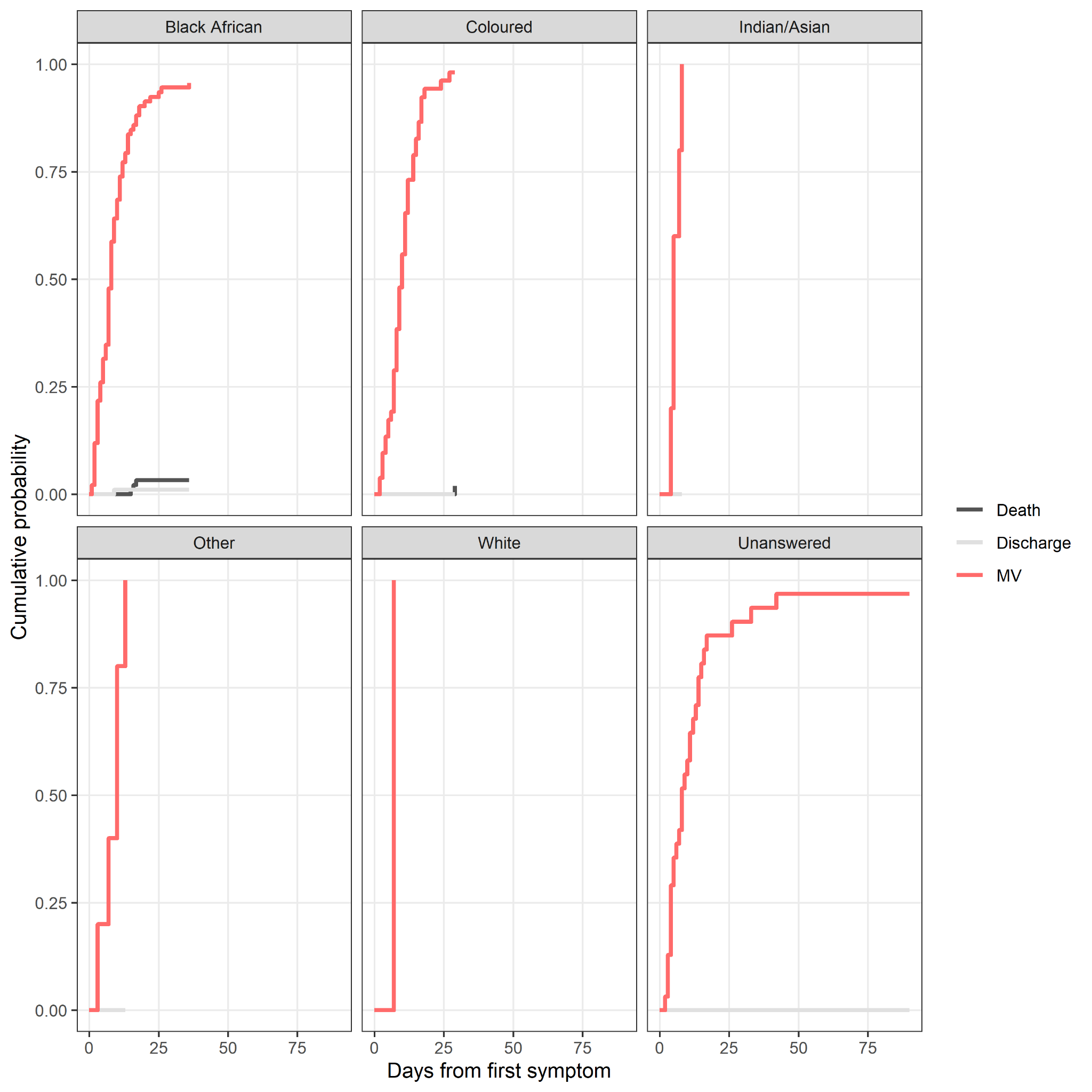


**Supplementary Figure 13: Timing of enrollment by country**


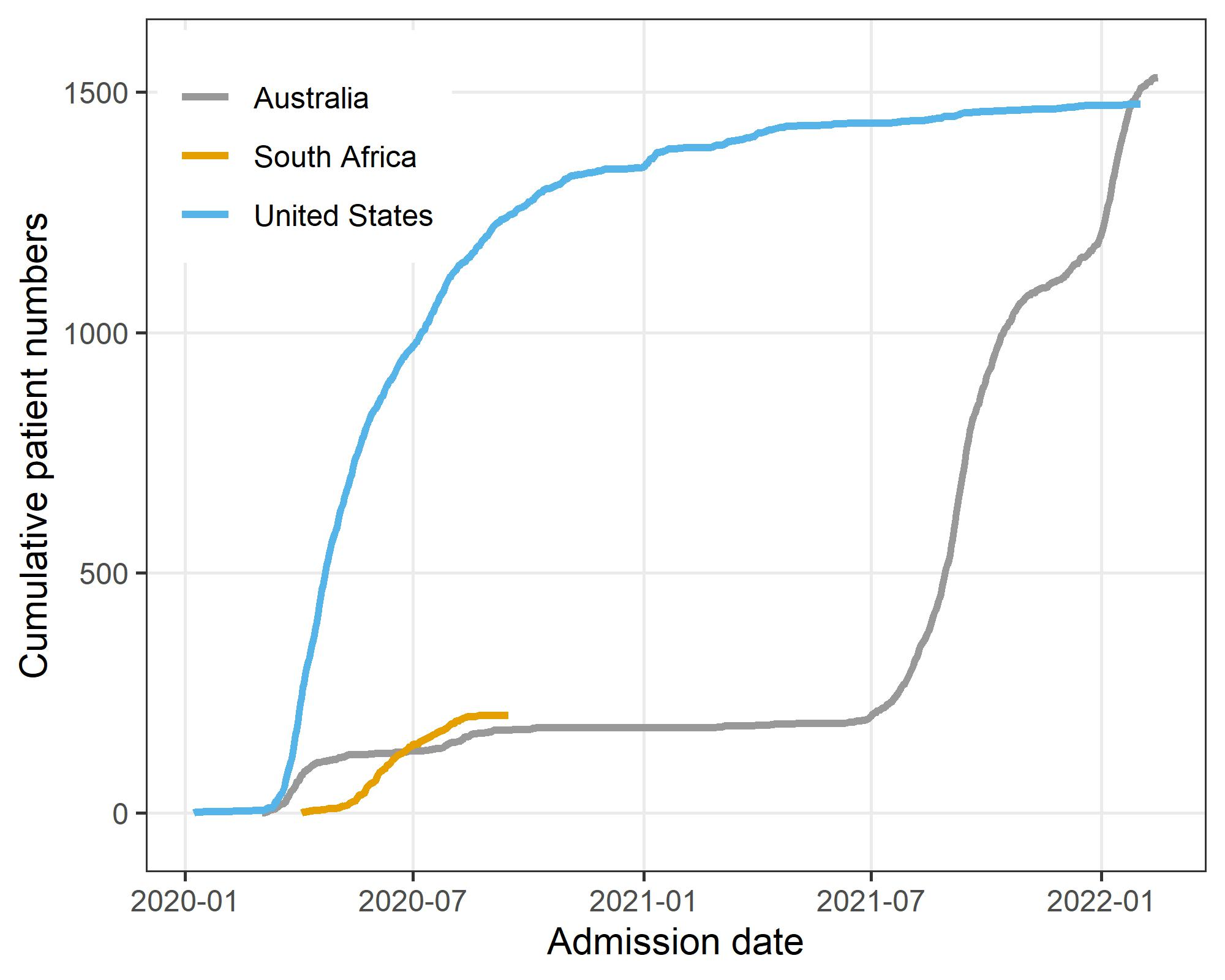


**Supplementary Figure 14A: Likelihood of Missing APACHE II by Ethnicity and Proportion of missing APACHE II by Site**

**
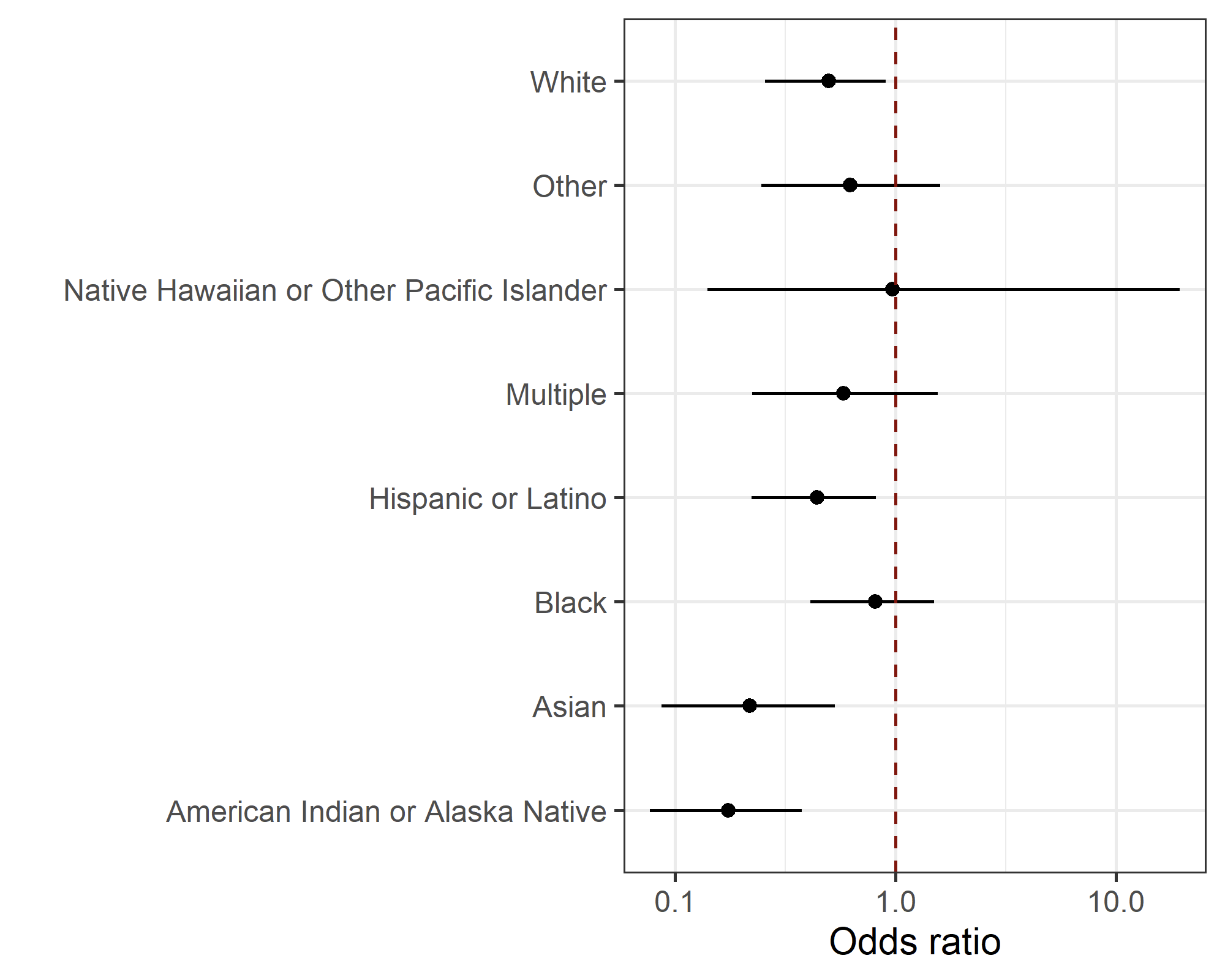
**

**Odds Ratio for Missing APACHE II by ethnicity, Groups to the left of 1.0 are less likely to have missing APACHE II Data (US Cohort).**

**Supplementary Figure 14B. Proportion of Missing APACHE II By Site (US Cohort)**

**
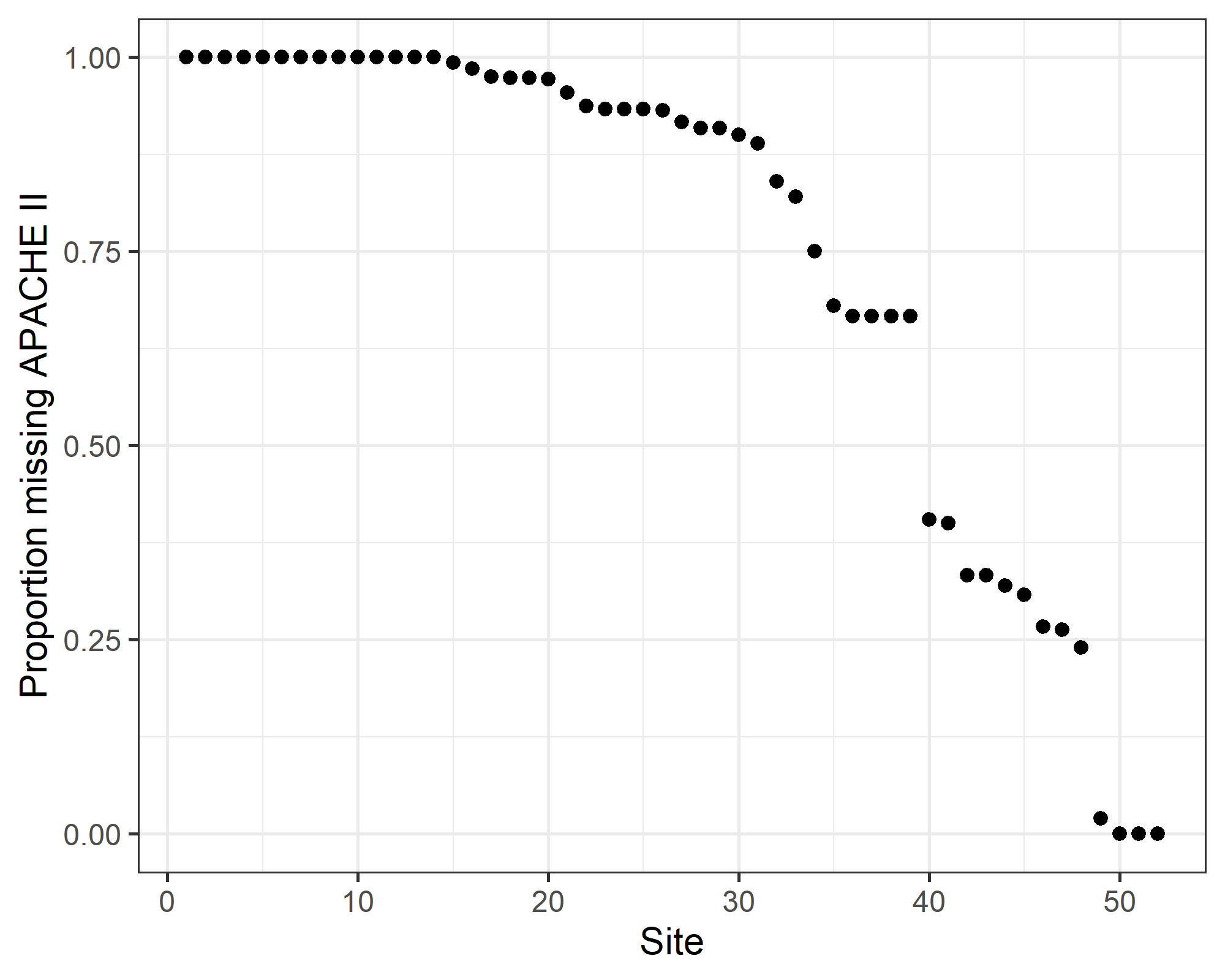
**
