# Supplementary material for: Race and ethnicity in the COVID-19 Critical Care Consortium: demographics, treatments, and outcomes, an international observational registry study
Source: Int J Equity Health. 2023 Dec 12;22:260. doi: 10.1186/s12939-023-02051-w (PMC10717789; doi:10.1186/s12939-023-02051-w)
Supplement: Supplementary file 2 — Additional file 2. [file 12939_2023_2051_MOESM2_ESM.docx]

**Contributors**

| **Prefix/First Name/Last Name** | **Site Name** |
| --- | --- |
| Tala Al-Dabbous Huda Alfoudri Mohammed Shamsah | Al Adan Hospital |
| Khadeejeh Alfroukh  Zinah Aqeel Abdulzahra Bairmani  Khalid Jehad Khalid  Salsabeel M.A. Abukhalaf | Al Ahli Hospital |
| Mohammed Maher Hadhoud | Al Menshawy General Hospital |
| Mohamed Fathi | Al-Hawari Center of Surgical Speciality |
| Hasan Alhouri | Al-Mouwasat University Hospital - Damascus |
| Dr Hamza Shahla | Al-Thawra Teaching Hospital in Albayda |
| Qamrah Alhadad Matly Hanan | Aljufrah Clinical Isolation Center |
| Subbarao Elapavaluru Ashley Berg Christina Horn | Allegheny General Hospital |
| Ahmed Reda Mohamed Elsayed Abdelhalim  Amro Essam Amer  Cinderella Omar Rageh Elnaggar  Ahmed Ayman Hassan  Ali Abdelaziz  Mohamed Abdelhalim  Yehia Samir Shaaban Aly Orabi | Alexandria Main University Hospital |
| Zinah A. Alaraji  Mo'nes R. Muhaisen  Lana Almasri  Dana Mustafa  Shaher Hamdan  Yousef Al-Saba'a  Zaina Dalloul  Mohammed Alkahlout  Hamza Jaber  Osama Aldabbourosama  Alaa Abdalfattah Abdalhadi | Alshifa Hospital, Gaza |
| Aliae AR Mohamed Hussein  Zarief Kamel Emad  Sarah Khaled  Nouralsabah Mohamed  Ebtisam Hassanin  Abdelhafeez Hamdi  May Gamal  Ahmed Emad  Abdelrahman Ragab  Mohammed G Azizeldin | Assiut University Hospital |
| Almthani Hamza  Alsarrah Ali Mohammed Omer  Asgad Osman Abdalla Fadl alla  Asia Atif Abdelrahman Abdallahrs  Aml Ahmed Eltayeb  Maali khalid mohamed abdalla Alhasan  Esraa Hassan Abdelgaum  Aya Mustafa Ahmed  Lamees Adil Abdulbaqi  Omer Abdullah Mohammedelhassan  Musaab Mohammed Mohammed Ahmed  Maha TagElser Mohammed Ali | Atbara Teaching Hospital |
| Yunis Mayasi | Avera McKennan Hospital & University Health Centre |
| Stephan Schroll | Barmherzige Bruder Regansburg |
| Dan Meyer Jorge Velazco Ludmyla Ploskanych Wanda Fikes Rohini Bagewadi Marvin Dao Haley White  Alondra Berrios Laviena  Ashley Ehlers Maysoon Shalabi-McGuire Trent Witt | Baylor Scott & White Health |
| Lorenzo Grazioli Luca Lorini | Bergamo Hospital |
| E. Wilson Grandin Jose Nunez Tiago Reyes | Beth Israel Deaconess Medical Centre |
| Diarmuid O’Briain Stephanie Hunter | Box Hill Hospital |
| Mahesh Ramanan Julia Affleck | Caboolture Hospital |
| Hemanth Hurkadli Veerendra  Sumeet Rai Josie Russell-Brown Mary Nourse | Canberra Hospital |
| Mark Joseph Brook Mitchell Martha Tenzer | Carilion Clinic |
| Ryuzo Abe | Chiba University Graduate School of Medicine |
| Hwa Jin Cho In Seok Jeong | Chonnam National University Hospital |
| Nadeem Rahman Vivek Kakar  Ahmed Tamimi  Diala Zabalawi  Mohamed Elhennawi  Praveen Ghisulal  Sadaf Malik | Cleveland Clinic- Abu Dhabi |
| Nicolas Brozzi | Cleveland Clinic - Florida |
| Omar Mehkri Sudhir Krishnan Abhijit Duggal Stuart Houltham | Cleveland Clinic - Ohio |
| Jerónimo Graf | Clinica Alemana De Santiago |
| Roderigo Diaz Roderigo Orrego  Camila Delgado Joyce González Maria Soledad Sanchez Michael Piagnerelli Josefa Valenzuela Sarrazin | Clinica Las Condez |
| A/Prof. Gustavo Zabert Lucio Espinosa Paulo Delgado Victoria Delgado | Clinica Pasteur National- University of Comahue |
| Diego Fernando Bautista Rincón Angela Maria Marulanda Yanten Melissa Bustamante Duque | Clinica Valle de Lilli |
| Daniel Brodie | Medical ICU, Columbia College of Physicians and Surgeons, New-York-Presbyterian Hospital, NY, NY, USA |
| Khaled Abouelmagd | Dr. Mohammad Alfagih Hospital |
| Alyaa Elhazmi Abdullah Al-Hudaib | Dr Sulaiman Alhabib Medical Group – Research Center, Riyadh, Saudi Arabia |
| Jeff Javidfar  Maria Callahan  Andy Dong  Charles Crepy D'Orleans | Emory University Healthcare System |
| M. Azhari Taufik  Elizabeth Yasmin Wardoyo Margaretha Gunawan Nurindah S Trisnaningrum Vera Irawany Muhammad Rayhan | Fatmawati Hospital |
| Mauro Panigada Antonio Pesenti Alberto Zanella Giacomo Grasselli Sebastiano Colombo  Chiara Martinet  Gaetano Florio | Fondazione IRCCS Policlinico of Milan (Fondazione IRCCS Ca' Granda Ospedale Maggiore Policlinico) |
| Massimo Antonelli Simone Carelli Domenico L. Grieco | Fondazione Policlinico Universitario Agostino Gemelli IRCCS |
| Motohiro Asaki | Fujieda Municipal General Hospital |
| Kota Hoshino | Fukuoka University |
| Leonardo Salazar  Mary Alejandra Mendoza Monsalve | Fundación Cardiovascular de Colombia |
| John Laffey Bairbre McNicholas David Cosgrave  Minha Atif  Fadi Qutishat  Caoimhe Laffey  Michaeal Van Der Walt | Galway University Hospitals |
| Joseph McCaffrey Allison Bone  Jemma Trickey  Michelle Horton  Michelle Horton  Stephanie Pearce  Tania Salerno | Geelong Hospital |
| Akram Mohamed  Salem Alhaddad  Baliad Bakeer  Shames Haitam  Laila Shalabi  Mohammed Abodina Ahmed | Gheryan Central Hospital |
| Yusuff Hakeem | Glenfield Hospital |
| James Winearls Mandy Tallott | Gold Coast University Hospital |
| David Thomson  Ivan Joubert Christel Arnold-Day  Jenna Piercy  Richard van Zyl Smit Malcom Miller Lisa Seymour  Francois van Heyningen  Gilbert Teyangesikayi  David Fredericks | Groote Schuur Hospital |
| Ali Ait Hssain Jeffrey Aliudin Al-Reem Alqahtani Khoulod Mohamed Ahmed Mohamed Darwin Tan Joy Villanueva Ahmed Zaqout  Ahmed Labib | Hamad General Hospital - Weill Cornell Medical College in Qatar |
| Ethan Kurtzman Arben Ademi Ana Dobrita Khadija El Aoudi Juliet Segura | Hartford HealthCare |
| Gezy Giwangkancana | Hasan Sadikin Hospital (Adult) |
| Shinichiro Ohshimo | Hiroshima University |
| Javier Osatnik | Hospital Alemán |
| Anne Joosten | Hospital Civil Marie Curie |
| Antoni Torres Minlan Yang Ana Motos | Hospital Clinic, Barcelona |
| Carlos Luna | Hospital de Clínicas |
| Francisco Arancibia | Hospital del Tórax |
| Virginie Williams Alexandre Noel | Hospital du Sacre Coeur (Universite de Montreal) |
| Nestor Luque | Hospital Emergencia Ate Vitarte |
| Marina Fantini | Hospital Mater Dei |
| Ruth Noemi Jorge García Enrique Chicote Alvarez | Hospital Nuestra Señora de Gracia |
| Anna Greti | Hospital Puerta de Hierro |
| Adrian Ceccato | Hospital Universitari Sagrat Cor |
| Angel Sanchez | Hospital Universitario Sant Joan d’Alacant |
| Ana Loza Vazquez | Hospital Universitario Virgen de Valme |
| Ferran Roche-Campo Diego Franch-Llasat | Hospital Verge de la Cinta de Tortosa |
| Divina Tuazon | Houston Methodist Hospital |
| Marcelo Amato Luciana Cassimiro Flavio Pola Francis Ribeiro Guilherme Fonseca | INCOR (Universidade de São Paulo) |
| Heidi Dalton Mehul Desai Erik Osborn Hala Deeb | INOVA Fairfax Hospital |
| Antonio Arcadipane Gennaro Martucci Giovanna Panarello Stefano Vitiello Claudia Bianco Giovanna Occhipinti Matteo Rossetti Raffaele Cuffaro | ISMETT |
| Nidhal Siddig | Jabra Hospital, Khartoum |
| Sung-Min Cho  Glenn Whitman | Johns Hopkins |
| Marwan El Sayed Walaa Mokhtar Eslam El-Shenawy | Kafr Elsheikh University Hospital |
| Hiroaki Shimizu Naoki Moriyama | Kakogawa Acute Care Medical Center |
| Jae-Burm Kim | Keimyung University Dong San Hospital |
| Nobuya Kitamura | Kimitsu Chuo Hospital |
| Johannes Gebauer | Klinikum Passau |
| Toshiki Yokoyama | Kouritu Tousei Hospital |
| Abdulrahman Al-Fares Sarah Buabbas Esam Alamad Fatma Alawadhi Kalthoum Alawadi  Mohamed Ahmed Khalefa  Nourah Ahmad Abdulaziz Al Ajeel  Mohammad Fathy Aly  Abdullah Al-Saleh  Abdullah Naanouh  Alaa Mohammed Elshourbgy  Abdulrahman Al-Fares  Mohamed Yousef Gad  Rania Mohamed ElRazaz  Ibrahim Khadadah  Ahmed Mohammed Almumin  Hala Altarakma  Hasan Albannay  Mohammed Kh Alsaleh  Mahmoud Saad Abdallah Radwan  Islam Ahmed Saadallah | Al-Amiri and Jaber Al-Ahmed Hospitals, Kuwait Extracorporeal Life Support Program |
| Hiro Tanaka | Kyoto Medical Centre |
| Satoru Hashimoto Masaki Yamazaki | Kyoto Prefectural University of Medicine |
| Tak-Hyuck Oh | Kyung Pook National University Chilgok Hospital |
| Mark Epler Cathleen Forney  Louise Kruse Jared Feister Joelle Williamson Katherine Grobengieser | Lancaster General Health |
| Eric Gnall Sasha Golden Mara Caroline  Timothy Shapiro Colleen Karaj Lisa Thome Lynn Sher Mark Vanderland Mary Welch Sherry McDermott | Lankenau Institute of Medical Research (Main Line Health) |
| Matthew Brain Sarah Mineall  Maria Unwin  Lixian Chen  Tarnya Trezise  Laurie McKeon | Launceston General Hospital |
| Dai Kimura | Le Bonheur Children’s Hospital |
| Luca Brazzi Gabriele Sales  Giorgia Montrucchio | Le Molinette Hospital (Ospedale Molinette Torino) |
| Tawnya Ogston | Legacy Emanuel Medical Center |
| Dave Nagpal Karlee Fischer | London Health Sciences Centre |
| Roberto Lorusso  Bas van Bussell  Maria Elena De Piero  Silvia Mariani | Maastricht University Medical Centre |
| Dr Rajavardhan Rangappa Dr Rajesh Mohan Shetty  Sujin Rai P  Argin Ganesan | Manipal Hospital Whitefield |
| Samar Tharwat | Mansoura University Hospital |
| Mariano Esperatti  Nora Angélica Fuentes  Maria Eugenia Gonzalez | Hospital Privado de Comunidad. Mar del Plata. Escuela Superior de Medicina. Universidad Nacional de  Mar del Plata |
| Diarmuid O’Briain | Maroondah Hospital |
| Edmund G. Carton | Mater Misericordiae University Hospital |
| Ayan Sen Amanda Palacios Deborah Rainey | Mayo Clinic College of Medicine |
| Gordan Samoukoviv Josie Campisi | McGill University Health Centre |
| Lucia Durham Emily Neumann Cassandra Seefeldt Octavio Falcucci Amanda Emmrich Jennifer Guy Carling Johns Kelly Potzner Catherine Zimmermann Angelia Espinal | Medical College of Wisconsin (Froedtert Hospital) |
| Nina Buchtele Michael Schwameis  Andrea Korhnfehl  Roman Brock  Thomas Staudinger | Medical University of Vienna |
| Stephanie-Susanne Stecher Michaela Barnikel  Sófia Antón  Alexandra Pawlikowski | Medical Department II, LMU Hospital Munich |
| Akram Zaaqoq Lan Anh Galloway Caitlin Merley | MedStar Washington Hospital Centre |
| Mohamed Muftah | Misurata Medical Center |
| Alistair Nichol | Monash University |
| Marc Csete Luisa Quesada Isabela Saba | Mount Sinai Medical Centre |
| Daisuke Kasugai Hiroaki Hiraiwa Taku Tanaka | Nagoya University Hospital |
| Eva Marwali Yoel Purnama Santi Rahayu Dewayanti Ardiyan Dafsah Arifa Juzar Debby Siagian | National Cardiovascular Center Harapan Kita, Jakarta, Indonesia |
| Yih-Sharng Chen | National Taiwan University Hospital |
| Amer Aldhalia | Nasar City Hospital for Insurance |
| Mark Ogino | Nemours Alfred I duPont Hospital for Children |
| Prashant Nasa Christina Matthew Nimisha Abdul Majeed | NMC Al Nahda Hospital Dubai |
| Wael Hafez | NMC Royal Hospital, Abu Dhabi |
| Indrek Ratsep Andra-Maris Post Piret Sillaots  Anneli Krund  Merili-Helen Lehiste  Tanel Lepik | North Estonia Medical Centre |
| Frank Manetta Effe Mihelis Iam Claire Sarmiento Mangala Narasimhan Michael Varrone | Northwell Health |
| Mamoru Komats | Obihiro-Kosei General Hospital |
| Julia Garcia-Diaz Catherine Harmon | Ochsner Clinic Foundation |
| S. Veena Satyapriya Amar Bhatt Nahush A. Mokadam Alberto Uribe Alicia Gonzalez Haixia Shi Johnny McKeown Joshua Pasek Juan Fiorda Marco Echeverria | Ohio State University Medical Centre |
| Rita Moreno | Oklahoma Heart Institute |
| Bishoy Zakhary | Oregon Health and Science University Hospital (OHSU) |
| Marco Cavana Alberto Cucino | Ospedale di Arco (Trento Hospital) |
| Giuseppe Foti Marco Giani Benedetta Fumagalli | Ospedale San Gerardo |
| Davide Chiumello Valentina Castagna | Ospedale San Paolo |
| Andrea Dell’Amore Paolo Navalesi | Padua University Hospital (Policlinico of Padova) |
| Hoi-Ping Shum | Pamela Youde Nethersole Eastern Hospital |
| Alain Vuysteke | Papworth Hospitals NHS Foundation Trust |
| Asad Usman Andrew Acker Benjamin Smood Blake Mergler Federico Sertic Madhu Subramanian Alexandra Sperry Nicolas Rizer | Penn Medicine (Hospital of the University of Pennsylvania) |
| Erlina Burhan  Menaldi Rasmin Ernita Akmal Faya Sitompul Navy Lolong Bhat Naivedh | Persahabatan General Hospital |
| Simon Erickson | Perth Children's Hospital |
| Peter Barrett David Dean Julia Daugherty | Piedmont Atlanta Hospital |
| Antonio Loforte | Policlinico di S. Orsola, Università di Bologna |
| Irfan Khan Mohammed Abraar Quraishi Olivia DeSantis | Presbyterian Hospital Services, Albuquerque |
| Ahmad Nasrallah | Prince Hamza Hospital- Amman |
| Dominic So Darshana Kandamby | Princess Margaret Hospital |
| Jose M. Mandei Hans Natanael | Prof Dr R. D. Kandou General Hospital - Paediatric |
| Eka YudhaLantang Anastasia Lantang | Prof Dr R. D R. D. Kandou General Hospital - Adult |
| Surya Oto Wijaya | Dr Sulianti Saroso Hospital |
| Anna Jung | Providence Saint John's Health Centre |
| George Ng Wing Yiu Ng | Queen Elizabeth Hospital, Hong Kong |
| Pauline Yeung Ng  Shu Fang | The University of Hong Kong |
| Alexis Tabah Megan Ratcliffe Maree Duroux | Redcliffe Hospital |
| Ahmed Alajeeli  Ali Tarhabat | Regdalin Hospital |
| Shingo Adachi Shota Nakao | Rinku General Medical Center (and Senshu Trauma and Critical Care Center) |
| Pablo Blanco Ana Prieto Jesús Sánchez | Rio Hortega University Hospital |
| Meghan Nicholson | Rochester General Hospital |
| Warwick Butt Alyssa Serratore Carmel Delzoppo | Royal Children’s Hospital |
| Pierre Janin Elizabeth Yarad | Royal North Shore Hospital |
| Richard Totaro Jennifer Coles | Royal Prince Alfred Hospital |
| Bambang Pujo | RSUD Soetomo |
| Robert Balk Andy Vissing Esha Kapania James Hays Samuel Fox Garrett Yantosh Pavel Mishin | Rush University, Chicago |
| Safia Adem | Sabha Medical Center |
| Saptadi Yuliarto Kohar Hari Santoso Susanthy Djajalaksana | Saiful Anwar Malang Hospital (Brawijaya University) (Paediatrics) |
| Arie Zainul Fatoni | Saiful Anwar Malang Hospital (Brawijaya University) (Adult) |
| Masahiro Fukuda | Saiseikai Senri Hospital |
| Keibun Liu | Saiseikai Utsunomiya Hospital |
| Paolo Pelosi Denise Battaglini  Chiara Robba | San Martino Hospital |
| Juan Fernando Masa Jiménez | San Pedro de Alcantara Hospital |
| Diego Bastos | Sao Camilo Cura D’ars |
| Sérgio Gaião | São João Hospital Centre, Porto |
| Desy Rusmawatiningtyas | Sardjito Hospital (Paediatrics) |
| Young-Jae Cho | Seoul National University Bundang Hospital |
| Su Hwan Lee | Severance Hospital |
| Tatsuya Kawasaki | Shizuoka Children’s Hospital |
| Laveena Munshi | Sinai Health Systems (Mount Sinai Hospital) |
| Pranya Sakiyalak Prompak Nitayavardhana | Siriraj Hospital |
| Mohamed Bashir Elagili  Talat Ahmed Abu Salem | Soug Althulatha Isolation Center |
| Tamara Seitz | Sozialmedizinisches Zentrum Süd – Kaiser-Franz-Josef-Spital |
| Rakesh Arora David Kent | St Boniface Hospital (University of Mannitoba) |
| Daniel Marino | St Christopher’s Hospital for Children |
| Swapnil Parwar Andrew Cheng Jennene Miller | St George Hospital |
| Shigeki Fujitani Naoki Shimizu | St Marianna Medical University Hospital |
| Jai Madhok Clark Owyang | Stanford University Hospital |
| Hergen Buscher Claire Reynolds | St Vincent’s Hospital |
| Abusalama Abdurraouf  Ali Abdulnasir Kredan  Abdurrahman Haddud | Swani Health Isolation Center |
| Saad Moharam | Tanta University Hospital |
| Olavi Maasikas AleksanBeljantsev Vladislav Mihnovits | Tartu University Hospital |
| Takako Akimoto Mariko Aizawa Kanako Horibe Ryota Onodera | Teine Keijinkai Hospital |
| Carol Hodgson Aidan Burrell Meredith Young | The Alfred Hospital |
| Timothy George | The Heart Hospital Baylor Plano, Plano |
| Kiran Shekar  Niki McGuinness Lacey Irvine | The Prince Charles Hospital |
| Brigid Flynn | The University of Kansas Medical Centre |
| Abdulrahman Almjersah | Tishreen University Hospital |
| Tomoyuki Endo | Tohoku Medical and Pharmaceutical University |
| Kazuhiro Sugiyama | Tokyo Metropolitan Bokutoh Hospital |
| Keiki Shimizu | Tokyo Metropolitan Medical Center |
| Eddy Fan Kathleen Exconde | Toronto General Hospital |
| Shingo Ichiba | Tokyo Women’s Medical University Hospital |
| Muhannud Binnawara | Tripoli Central Hospital |
| Hussein Embarek | Tripoli University Hospital |
| Leslie Lussier | Tufts Medical Centre (and Floating Hospital for Children) |
| Gösta Lotz | Universitätsklinikum Frankfurt (University Hospital Frankfurt) (Uniklinik) |
| Maximilian Malfertheiner Lars Maier Esther Dreier | Universitätsklinikum Regensburg (Klinik für Innere Medizin II) |
| Neurinda Permata Kusumastuti | University Airlangga Hospital (Paediatric) |
| Colin McCloskey Al-Awwab Dabaliz Tarek B Elshazly Josiah Smith | University Hospital Cleveland Medical Centre (UH Cleveland Hospital) |
| Konstanty S. Szuldrzynski Piotr Bielański | University Hospital in Krakow |
| Yusuff Hakeem | University Hospitals of Leicester NHS Trust (Glenfield Hospital) |
| Keith Wille | University of Alabama at Birmingham Hospital (UAB) |
| Srinivas Murthy | University of British Columbia |
| Ken Kuljit S. Parhar Kirsten M. Fiest  Cassidy Codan Anmol Shahid | University of Calgary (Peter Lougheed Centre, Foothills Medical Centre, South Health Campus and Rockyview General Hospital) |
| Mohamed Fayed Timothy Evans Rebekah Garcia Ashley Gutierrez Hiroaki Shimizu | University of California, San Francisco-Fresno Clinical Research Centre |
| Tae Song Rebecca Rose | University of Chicago |
| Suzanne Bennett Denise Richardson | University of Cincinnati Medical Centre |
| Giles Peek | University of Florida |
| Lovkesh Arora Kristina Rappapport Kristina Rudolph Zita Sibenaller Lori Stout Alicia Walter | University of Iowa |
| Daniel Herr Nazli Vedadi | University of Maryland - Baltimore |
| Robert Bartlett | University of Michigan Medical Center |
| Antonio Pesenti | University of Milan |
| Shaun Thompson Julie Hoffman Xiaonan Ying Bailey Williams Emely Sanchez Chika Akwani | University of Nebraska Medical Centre |
| Ryan Kennedy | University of Oklahoma Health Sciences Centre (OU) |
| Muhammed Elhadi | Faculty of Medicine, University of Tripoli |
| Matthew Griffee  Mary Mone Anna Ciullo Yuri Kida | University of Utah Hospital |
| Ricard Ferrer Roca JordI Riera Sofia Contreras Cynthia Alegre | Vall d'Hebron University Hospital, Barcelona |
| Christy Kay Irene Fischer Elizabeth Renner | Washington University in St. Louis/ Barnes Jewish Hospital |
| Hayato Taniguci | Yokohama City University Medical Center |
| James Lee Daniel Plotkin Barbara Wanjiru Citarella Laura Merson | ISARIC, Centre for Tropical Medicine and Global Health, University of Oxford, Oxford, UK |

**Collaborators**

| **Prefix/First Name/Last Name** | **Site Name** |
| --- | --- |
| Emma Hartley | Aberdeen Royal Infirmary (Foresterhill Health Campus) |
| Bastian Lubis | Adam Malik Hospital |
| Takanari Ikeyama | Aichi Childrens Health and Medical Center |
| Alshaymaa Mortada | Ain Shams University |
| Ameen Alhamad | Aleppo University Hospital |
| Ahmed Mechi | Al-Sader Teaching Hospital, Al-Najaf |
| Islam Mohsen Ali Mohamed Hassan Nadar | Al Salam Specialized Hospital |
| Mohammed Saleh Alyasiri | Alshifa Center Medical City |
| Muhammed Zainab Alghali Elsaid | Alshuhada Hospital |
| Balu Bhaskar | American Hospital |
| Jae-Seung Jung | Anam Korea University Hospital |
| Shay McGuinness | Auckland City Hospital |
| Glenn Eastwood | Austin Hospital |
| Sandra Rossi Marta  Fabio Guarracino | Azienda Ospedaliero Universitaria Parma |
| Stacy Gerle | Banner University Medical Centre |
| Emily Coxon | Baptist Health Louisville |
| Bruno Claro | Barts Hospital |
| Wafa Aldressi | Benghazi Medical Centre |
| Mahmoud Eleisawy | Benha University Hospital |
| Hasnaa Osama | Beni-Suef University Hospital |
| Daniel Loverde | Billings Clinic |
| Namrata Patil | Brigham and Women’s Hospital |
| Vieri Parrini | Borgo San Lorenzo Hospital |
| Angela McBride | Brighton and Sussex Medical School |
| Kathryn Negaard | Brooke Army Medical Centre |
| Angela Ratsch | Bundaberg Hospital |
| Ahmad Abdelaziz | Cairo University Hospital |
| Juan David Uribe | Cardio VID |
| Adriano Peris | Careggi Hospital |
| Mark Sanders | Cedar Park Regional Medical Center |
| Dominic Emerson | Cedars-Sinai Medical Centre |
| Muhammad Kamal | Cengkareng Hospital |
| Hamza Faida | Centre Hospitalier Universitaire Ibn Sina Rabat |
| Pedro Povoa | Centro Hospitalar de Lisboa |
| Roland Francis | Charite-Univerrsitatsmedizi n Berlin |
| Ali Cherif | Charles Nicolle University Hospital |
| Sunimol Joseph | Children’s Health Ireland (CHI) at Crumlin |
| Matteo Di Nardo | Children’s Hospital Bambino Gesù |
| Micheal Heard | Children's Healthcare of Atlanta – Egleston Hospital |
| Kimberly Kyle | Children's Hospital – Los Angeles |
| Ray A Blackwell | Christiana Care Health System's Centre for Heart and Vascular Health |
| Amel OUYAHIA | CHU - Chu Saadna Abdenour De Sétif - Sétif |
| Michael Piagnerelli  Patrick Biston | CHU de Charleroi |
| Hye Won Jeong | Chungbuk National University Hospital |
| Reanna Smith | Cincinnati Children's |
| Yogi Prawira | Cipto Mangunkusumo Hospital |
| Giorgia Montrucchio | Città della Salute e della Scienza Hospital – Turin, Italy |
| Arturo Huerta Garcia | Clínica Sagrada Família |
| Nahikari Salterain | Clinica Universidad de Navarra |
| Bart Meyns | Collaborative Centre Department Cardiac Surgery, UZ Leuven |
| Muhammed Elnasser | Damascus Hospital |
| Marsha Moreno | Dignity Health Medical Group- Dominican |
| Rajat Walia | Dignity Health St. Joseph's Hospital and Medical Center (SJHMC) |
| Amit Mehta | Doernbecher Children’s Hospital |
| Annette Schweda | Donaustauf Hospital |
| Melissa Williams | Duke University Hospital (Durham) |
| Emad Amkhatirah | Elmarj Teaching Hospital |
| Kyung Hoon Kim | Eunpyeung St Mary's Hospital |
| Alexandra Assad | Fluminense Federal University |
| Estefania Giraldo | Fundación Clinica Shaio (Shaio Clinic) |
| Wojtek Karolak | Gdansk Medical University |
| Martin Balik | General University Hospital |
| Elizabeth Pocock | George Washington University Hospital |
| Akram Mohamed | Gharyan Central Hospital |
| Evan Gajkowski | Giesinger Medical Centre |
| Mohamed Bedair | Giza International Hospital |
| Kanamoto Masafumi | Gunma University Graduate School of Medicine |
| Nicholas Barrett | Guy's and St Thomas NHS Foundation Trust Hospital |
| Yoshihiro Takeyama | Hakodate City Hospital |
| Sunghoon Park | Hallym University Sacred Heart Hospital |
| Faizan Amin | Hamilton General Hospital |
| Fina Meilyana Andriyani | Hasan Sadikin Hospital (Paediatric) |
| Serhii Sudakevych | Heart Institute Ministry of Health of Ukraine |
| Janos Schnur | Heim Pál National Pediatric Institute |
| Angela Ratsch | Hervey Bay Hospital |
| Magdalena Vera | Hospital Clinico de la Pontificia Universidad Catolica |
| Rodrigo Cornejo | Hospital Clinico de la Universidad de Chile |
| Patrícia Schwarz  Ana Carolina Mardini | Hospital de Clínicas de Porto Alegre |
| Thais de Paula | Hospital Felicio Rocho |
| Ary Serpa Neto | Hospital Israelita Albert Einstein |
| Andrea Villoldo | Hospital Privado de Comunidad |
| Alexandre Siciliano Colafranceschi | Hospital Pro Cardíaco |
| Alejandro Ubeda Iglesias | Hospital Punta de Europa |
| Juan Granjean | Hospital Regional de Valdivia |
| Lívia Maria Garcia Melro  Giovana Fioravante Romualdo | Hospital Samaritano Paulista |
| Diego Gaia | Hospital Santa Catarina |
| Helmgton Souza | Hospital Santa Marta |
| Filomena Galas | Hospital Sirio Libanes |
| Rafael Máñez Mendiluce | Hospital Universitario de Bellvitge |
| Alejandra Sosa | Hospital Universitario Esperanza (Universidad Francisco Marroquin) |
| Ignacio Martinez | Hospital Universitario Lucus Augusti |
| Hiroshi Kurosawa | Hyogo Prefectural Kobe Children's Hospital |
| Mohammad Badr Almoshantaf | Ibn Al-Nafees Hospital |
| Juan Salgado | Indiana University Health |
| Beate Hugi-Mayr | Inselspital University Hospital |
| Eric Charbonneau | Institut Universitaire de Cardiologie et de Pneumologie de Quebec - Universite Laval |
| Vitor Salvatore Barzilai | Instituto de Cardiologia do Distrito Federal - ICDF |
| Veronica Monteiro | Instituto de Medicina Integral . Fernando Figueira (IMIP) |
| Rodrigo Ribeiro de Souza | Instituto Goiano de Diagnostico Cardiovascular (IGDC) |
| Michael Harper | INTEGRIS Baptist Medical Center |
| Hiroyuki Suzuki | Japan Red Cross Maebashi Hospital |
| Celina Adams | John C Lincoln Medical Centre |
| Jorge Brieva | John Hunter Hospital |
| Almu'atasim Khamees | Jordan University Hospital |
| Fadi Graige | Kalamoon Hospital |
| Moh Supriatna | Kariadi Hospital Semarang |
| George Nyale | Kenyatta National Hospital (KNH) |
| Faisal Saleem Eltatar | King Abdullah Medical City |
| Jihan Fatani | King Abdullah Medical City Specialist Hospital |
| Husam Baeissa | King Abdullah Medical Complex |
| Ayman AL Masri | King Salman Hospital NWAF |
| Ahmed Rabie | King Saud Medical City |
| Mok Yee Hui | KK Women's and Children's Hospital |
| Masahiro Yamane | KKR Medical Center |
| Hanna Jung | Kyung Pook National University Hospital |
| Ayorinde Mojisola Margaret | Lagos University Teaching Hospital |
| Newell Nacpil | Lung Center of the Philippines |
| Katja Ruck | Luxembourg Heart Center |
| Rhonda Bakken | M Health Fairview |
| Claire Jara | Maine Medical Centre (Portland Maine) |
| Tim Felton | Manchester University NHS Foundation Trust - Wythenshawe |
| Lorenzo Berra | Massachusetts General Hospital |
| Bobby Shah | Medanta Hospital |
| Arpan Chakraborty | Medica Super speciality Hospital |
| Monika Cardona | Medical University of South Carolina |
| Gerry Capatos | Mediclinic Parkview Hospital Dubai |
| Bindu Akkanti | Memorial Hermann - Texas Medical Centre |
| Abiodun Orija | Memorial Regional Hospital (Hollywood Florida) |
| Harsh Jain | Mercy Hospital of Buffalo |
| Asami Ito | Mie University Hospital |
| Brahim Housni | Mohammed VI University Hospital |
| Sennen Low | National Centre for Infectious Diseases |
| Koji Iihara | National Cerebral and Cardiovascular Center |
| Joselito Chavez | National Kidney and Transplant Institute |
| Kollengode Ramanathan | National University Hospital, Singapore |
| Gustavo Zabert | National University of Comahue |
| Krubin Naidoo | Nelson Mandela Children's Hospital |
| Ian Seppelt | Nepean Hospital |
| Marlice VanDyk  Sarah MacDonald | Netcare Unitas ECMO Centre |
| Shingo Ichiba | Nippon Medical School Hospital |
| Randy McGregor | Northwestern Medicine |
| Teka Siebenaler | Norton Children's Hospital |
| Hannah Flynn | Novant Health (NH) Presbyterian Medical Centre |
| Kristi Lofton | Ochsner LSA Health Shreveport |
| Toshiyuki Aokage | Okayama University Hospital |
| Bakar Kvirkvelia | Open Heart 5^th^ Clinical Hospital, Tbilisi |
| Kazuaki Shigemitsu | Osaka City General Hospital |
| Andrea Moscatelli | Ospedale Gaslini |
| Giuseppe Fiorentino | Ospedali dei Colli |
| Matthias Baumgaertel | Paracelsus Medical University Nuremberg |
| Serge Eddy Mba | Parirenyatwa General Hospital |
| Jana Assy | Pediatric and Neonatal Cardiac Intensive Care at the American University |
| Amelya Hutahaean | Pelni Hospital |
| Holly Roush | Penn State Heath S. Hershey Medical Centre |
| Kay A Sichting | Peyton Manning Children's Hospital |
| Francesco Alessandri | Policlinico Umberto, Sapienza University of Rome |
| Debra Burns | Presbyterian Hospital, New York/ Weill Cornell Medical Centre |
| Taha Husayn Alkhubouli | Preventive Medicine Hospital |
| Ahmad Nasrallah | Prince Hamza Hospital- Amman |
| Ahmed Rabie | Prince Mohammed bin Abdulaziz Hospital |
| Gavin Salt | Prince of Wales |
| Carl P. Garabedian | Providence Sacred Heart Children's Hospital |
| Jonathan Millar  Malcolm Sim | Queen Elizabeth II University Hospital |
| Adrian Mattke | Queensland Children’s Hospital |
| Danny McAuley | Queens University of Belfast |
| Jawad Tadili | Rabat University Hospital |
| Tim Frenzel | Radboud University Medical Centre |
| Amro Abuleil | Rafidia Surgical Hospital |
| Yaron Bar-Lavie | Rambam Hospital |
| Aaron Blandino Ortiz | Ramón y Cajal University Hospital |
| Jackie Stone | Rapha Medical Centre |
| Alexis Tabah | Redcliffe Hospital |
| Antony Attokaran | Rockhampton Hospital |
| Michael Farquharson | Royal Adelaide Hospital |
| Brij Patel | Royal Brompton & Harefield NHS Foundation Trust |
| Derek Gunning | Royal Columbian Hospital |
| Kenneth Baillie | Royal Infirmary Edinburgh |
| Pia Watson | Sahlgrenska University Hospital |
| Kenji Tamai | Saiseikai Yokohamashi Tobu Hospital |
| Gede Ketut Sajinadiyasa  Dyah Kanyawati | Sanglah General Hospital |
| Marcello Salgado | Santa Casa de Misericordia de Juiz de Fora |
| Assad Sassine | Santa Casa de Misericórdia de Vitoria |
| Bhirowo Yudo | Sardjito Hospital |
| Scott McCaul | Scripps Memorial Hospital La Jolla |
| Bongjin Lee | Seoul National University Children's Hospital |
| Sang Min Lee | Seoul National University Hospital |
| Arnon Afek | Sheba Medical Center |
| Shimaa E Fattouh | Sherbin General Hospital |
| Yoshiaki Iwashita | Shimane University Hospital |
| Hammad Fadlalmola | Soba University Hospital |
| Bambang Pujo Semedi  Neurinda Permata Kusumastuti | Soetomo General Hospital (FK UNAIR) |
| Noureldin Mohamed Mansour | Souad Kafafi University Hospital |
| Jack Metiva | Spectrum Health Western Governors University |
| Nicole Van Belle | St. Antonius Hospital |
| Ignacio Martin-Loeches | St James’s University Hospital |
| Dr Mohammed Al-Sadawi | Stony Brook University |
| Cenk Kirakli | Suat Seren Chest Diseases and Surgery Practice and Training Centre |
| Al-Touny Shimaa | Suez Canal University Hospitals |
| Lenny Ivatt | Swansea Hospital |
| Chia Yew Woon | Tan Tock Seng Hospital |
| Hyun Mi Kang | The Catholic University of Seoul St Mary Hospital |
| Timothy Smith | The Christ Hospital |
| Erskine James | The Medical Centre Navicent Health |
| Nawar Al-Rawas | Thomas Jefferson University Hospital |
| Yudai Iwasaki | Tohoku University |
| Hamza Ashour | Traghen Hospital |
| Kenny Chan King-Chung | Tuen Mun Hospital |
| Vadim Gudzenko | UCLA Medical Centre (Ronald Regan) |
| Beate Hugi-Mayr | Universitätsspital Bern, Universitätsklinik für Herz- und Gefässchirurgie |
| Fabio Taccone | Universite Libre de Bruxelles |
| Fajar Perdhana | University Airlangga Hospital (Adult) |
| Yoan Lamarche | University de Montreal (Montreal Heart Institute) |
| Joao Miguel Ribeiro | University Hospital CHLN |
| Nikola Bradic | University Hospital Dubrava |
| Klaartje Van den Bossche | University Hospital Leuven |
| Oude Lansink | University Medical Center Groningen |
| Gurmeet Singh | University of Aberta (Mazankowski Heart Institute) |
| Gerdy Debeuckelaere | University of Antwerp |
| Henry T. Stelfox | University of Calgary and Alberta Health Services |
| Cassia Yi | University of California at San Diego |
| Jennifer Elia | University of California, Irvine |
| Thomas Tribble | University of Kentucky Medical Center |
| Shyam Shankar | University of Missouri |
| Raj Padmanabhan | University of Pittsburgh Medical Centre |
| Bill Hallinan | University of Rochester Medical Centre (UR Medicine) |
| Luca Paoletti | University of South Carolina |
| Yolanda Leyva | University of Texas Medical Branch |
| Tatuma Fykuda | University of the Ryukyus |
| Jenelle Badulak | University of Washington in Seattle |
| Jillian Koch | University of Wisconsin & American Family Children's Hospital |
| Lisa Janowaik | UTHealth (University of Texas) |
| Amy Hackman | UT Southwestern |
| Deb Hernandez | Valley Children's Hospital (Madera) |
| Jennifer Osofsky | Vassar Brothers Medical Center (VBMC) |
| Katia Donadello | Verona Integrated University Hospital |
| Aizah Lawang | Wahidin Sudirohusodo Hospital |
| Josh Fine | WellSpan Health - York Hospital |
| Benjamin Davidson | Westmead Hospital |
| Andres Oswaldo Razo Vazquez | Yale New Haven Hospital |
| Ibrahim Abdehaleem | Zagazig University Hospital |
